# Recurrent palaeo-wildfires in a Cisuralian coal seam: A palaeobotanical view on high-inertinite coals from the Lower Permian of the Paraná Basin, Brazil

**DOI:** 10.1371/journal.pone.0213854

**Published:** 2019-03-14

**Authors:** José Rafael W. Benicio, André Jasper, Rafael Spiekermann, Luciane Garavaglia, Etiene Fabbrin Pires-Oliveira, Neli Teresinha Galarce Machado, Dieter Uhl

**Affiliations:** 1 Programa de Pós-Graduação em Ambiente e Desenvolvimento (PPGAD), Universidade do Vale do Taquari–Univates, Lajeado, Rio Grande do Sul, Brazil; 2 Laboratório de Paleobotânica e Evolução de Biomas do Museu de Ciências Naturais da Univates (LPEB/MCN/Univates), Lajeado, Rio Grande do Sul, Brazil; 3 Senckenberg Forschungsinstitut und Naturmuseum, Frankfurt am Main, Germany; 4 Centro Tecnológico de Carvão Limpo, SACT, Criciúma, Santa Catarina, Brazil; 5 Laboratório de Paleobiologia, Universidade Federal do Tocantins, Porto Nacional, Tocantins, Brazil; Khalifa University of Science and Technology, UNITED ARAB EMIRATES

## Abstract

Distribution and abundance of charcoal in coal seams (in form of pyrogenic macerals of the inertinites group) have been considered as a reliable tool to interpret the local and regional palaeo-wildfire regimes in peat-forming depositional environments. Although the occurrence of inertinites is globally well documented for the Late Palaeozoic, the description of palaeobotanical evidence concerning the source plants of such charcoal is so far largely missing. In the present study, we provide the first detailed analysis of macro-charcoal preserved in the Barro Branco coal seam, Rio Bonito Formation, Cisuralian of the Paraná Basin, Santa Catarina State, Brazil. Charcoal, in form of macro-charcoal and inertinites, was documented in all the six coal-bearing strata that compose the succession, confirming the occurrence of recurrent palaeo-wildfires during its deposition. Reflectance values indicated a mean charring temperature reaching ~515°C (and up to 1,045°C in excess) and the macro-charcoal exhibits anatomical features of secondary xylem of *Agathoxylon*. Combination of results derived from palaeobotanical and petrological data demonstrates that gymnosperm-dominated vegetation was repeatedly submitted to fire events and reinforced the hypothesis that Gondwanan mires were high-fire systems during the Cisuralian.

## Introduction

The distribution and abundance of fossil charcoal in coal seams, which is well documented for the Late Palaeozoic in form of pyrogenic inertinites [1], is a reliable tool to understand palaeo-wildfire events and interpret certain palaeoenvironmental conditions of the original peat-forming systems [2, 3]. However, compared to coal petrological studies on inertinites, studies on palaeo-wildfire records based on palaeobotanical evidence (like anatomical analysis of charcoal) are still scarce for Gondwana, where entire Permian lithostratigraphic units have not been examined so far [1, 4, 5, 6].

For the Paraná Basin in Brazil, known macro-charcoal occurrences are mostly restricted to the southernmost levels of the Triunfo Member, a basal package of the Early Permian Rio Bonito Formation [4, 7, 8]. Except for some reports for the Bonito coal seam [4, 9], so far, the younger Paraguaçu and Siderópolis Members of the Rio Bonito Formation have not been studied in detail to examine the presence of macro-charcoal remains.

In the present study, we provide the first detailed description of macro-charcoal from the Barro Branco coal seam, Siderópolis Member of the Rio Bonito Formation. Besides providing anatomical descriptions and interpreting taxonomical affinities, we compared the presence of macro-charcoal remains to coal maceral contents in each studied carbonaceous level, aiming to demonstrate the importance of combining methods for a reliable reconstruction of palaeo-wildfire occurrences in peat-forming vegetation through the Late Palaeozoic in Gondwana.

## Geological context

**Fig 1:**
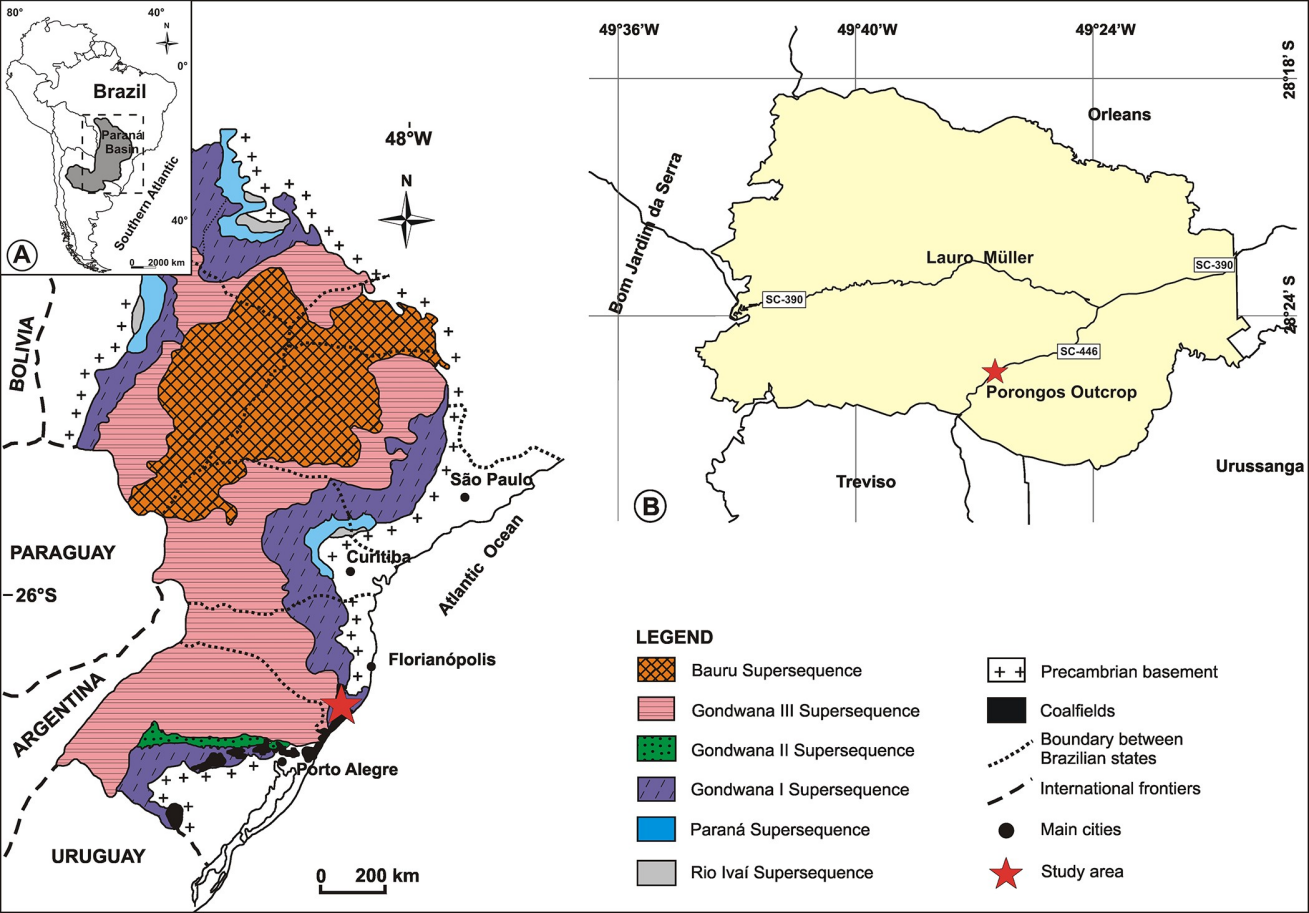
Location of the Paraná Basin and Porongos outcrop. A) Simplified geological map of the Paraná Basin, showing the major tectonic elements and indicating the studied locality (adapted from [11]). B) Geographical position of the Porongos outcrop and adjacent areas.

The Paraná Basin (Fig 1A) is an extensive intracratonic sedimentary basin covering ~1,700,000 km^2^, of the central portion of South America. The basin floor subsidence, associated with Palaeozoic and Mesozoic sea-level variations, generated six second-order super sequences deposited from the Ordovician to the Late Cretaceous. The major Palaeozoic transgressive-regressive cycles are exposed in the Rio Ivaí (Ordovician-Silurian), Paraná (Devonian), and Gondwana I (Carboniferous-Early Triassic) supersequences, including the Cisuralian coal-bearing strata of the Rio Bonito Formation [10, 11].

The Rio Bonito Formation has been formally divided into three lithostratigraphic members, named from the base to the top as Triunfo Member, Paraguaçu Member and Siderópolis Member [12]. The Triunfo Member is composed of coastal and fluvial sandstones as well as coal deposits, the Paraguaçu Member comprises mudstones, coal deposits and fine-grained marine sandstones, and the Siderópolis Member consists of coastal and fluvial sandstones and coal deposits [12, 13].

**Fig 2:**
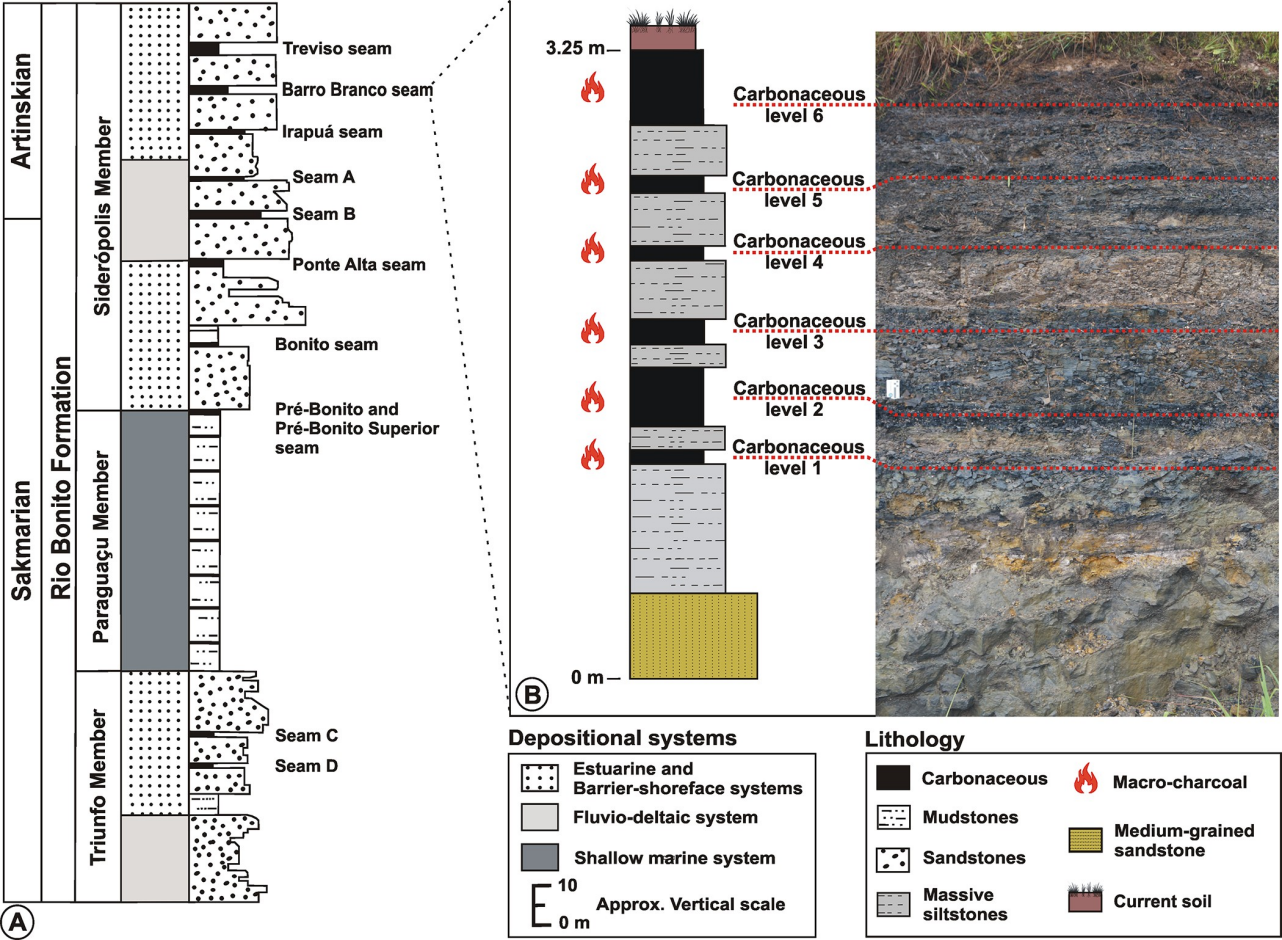
Stratigraphy of the Rio Bonito Formation and stratigraphic column of the Porongos outcrop. A) General stratigraphical framework of the Rio Bonito Formation in Santa Catarina state (adapted from [15]). B) Stratigraphical column of the Porongos outcrop showing the six macro-charcoal bearing layers.

At the south-eastern part of the distributional area of the Rio Bonito Formation, the Siderópolis Member contains the thickest coal seams, which are informally named, from the base to the top, as Bonito, Ponte Alta (A and B), Irapuá, Barro Branco and Treviso coal seams [14, 15] (Fig 2A). These were formed in an estuarine-barrier shore-face depositional context, and peat accumulation occurred during high stand system tract (Bonito), low stand system tract (Ponte Alta A and B) and transgressive system tract (Irapuá, Barro Branco and Treviso) [15].

The Barro Branco coal seam is composed of a coal layer (informally named as *Banco*) at the base, siltstones and sandstones, interbedded with thin coal layers (informally named as *Coringa* or *Quadração*) at the middle, and a carbonaceous layer of variable thickness (informally named as *Forro*) at the top. The seam has been extensively exploited and has a wide and continuous geographical distribution, with an average thickness ranging from 1.66 to 2.27 m. However, its net coal contents are less thick (0.47 to 1.40 m) due to interbedded levels of shales and siltstones [15].

Considering sequence stratigraphy as well as palaeontological and lithostratigraphic criteria, the Rio Bonito Formation was divided into two third-order sequences (LPTS-3 and LPTS-4) [13]. The Siderópolis Member was included in the LPTS-4 sequence and an Artinskian age for the coal seams was suggested [13].

## Material and methods

The material was collected at the Porongos outcrop, located in the municipality of Lauro Müller, Santa Catarina state, Brazil, at the coordinates 28^o^ 25’ 21.4” S 49^o^ 26’ 24.0” W (Fig 1B). Overlying a medium-grained sandstone (0.40 m), the Barro Branco coal seam is exposed as a 2.85 m succession consisting of 6 carbonaceous levels, each only a few centimetres thick (named here as Carbonaceous 1–6) interbedded by 5 medium-grey siltstones (Fig 2B).

In the field, 10 hand samples were collected from each outcropping level and taken to the *Laboratório de Paleobotânica e Evolução de Biomas*, *Museu de Ciências*, *Universidade do Vale do Taquari–Univates (LPEB/MCN/Univates)* for analysis under stereomicroscope (Zeiss Stemi 2000–C). Plant remains exhibiting characteristics of macro-charcoal (≥ 2.0 mm; black colour; silky lustre and; black streak on touch) [16, 17, 18], were mechanically extracted with the aid of forceps and needles. Subsequently, the plant remains were mounted on stubs with adhesive tabs, coated with gold, and investigated under Scanning Electron Microscope (SEM–Zeiss EVO LS15) at the *Parque Científico e Tecnológico do Vale do Taquari (TECNOVATES–Univates)*.

Anatomical features were measured with the use of the software ImageJ [19] from digital images. All the macro-charcoal and inertinite bearing rock samples collected are stored in the *LPEB/MCN/Univates* Palaeobotanical Collection under accession numbers PbUMCN1163–1168.

Maceral and reflectance analyses were conducted on the macro-charcoal containing samples at the *Laboratório de Análise de Carvão e Rochas Geradoras de Petróleo* of the *Universidade Federal do Rio Grande do Sul (UFRGS)* by using the standard preparations for optical analysis [20]. The maceral analysis was based on 500 observation points [21] and classified according to the International Committee for Coal Petrology [22, 23, 24] standards. The reflectance values were determined according to [25] and charring temperature was estimated based on the inertinite reflectance average, calculated by the mathematical formula [^o^C = 184 + 118 x (light reflectance %)] [26].

## Results

### Macro-charcoal overview

**Fig 3:**
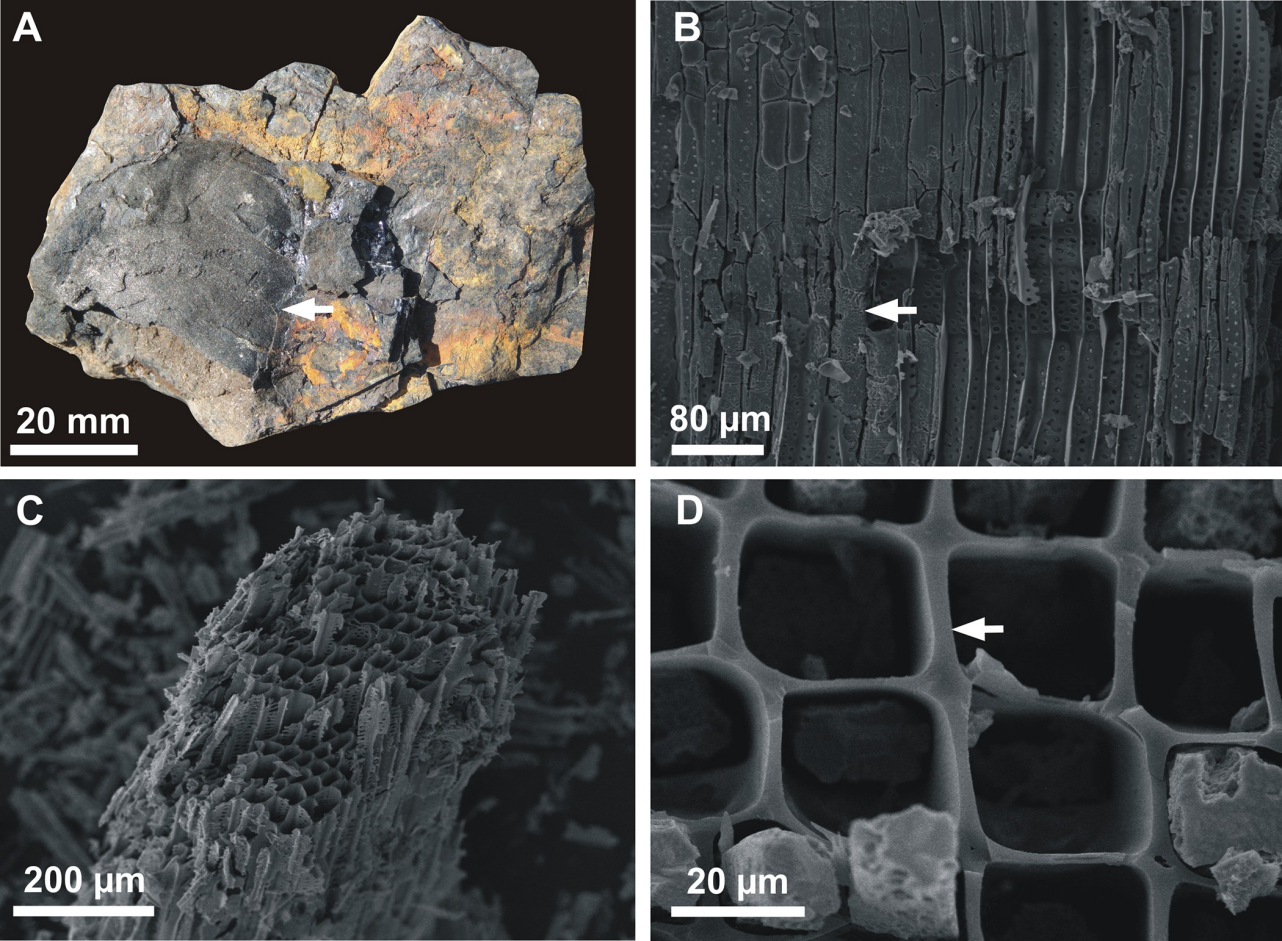
Overview of the macro-charcoal remains from Porongos outcrop. A) Fragment showing slightly abraded edges (sample PBUMCN 1167). B) Macro-charcoal impregnated by pyrite (fragment extracted from PBUMCN 1165). C) Well-preserved anatomical details (fragment extracted from PBUMCN 1168). D) Homogenized cell walls (fragment extracted from PBUMCN 1165).

Macro-charcoal, ranging between 3–49 mm in width and 5–111 mm in length, was recovered from each of the six carbonaceous levels exposed at the Porongos outcrop (Fig 2B). The fragments show slightly abraded edges (Fig 3A) and are impregnated by pyrite (Fig 3B). Compression is slight and no *Bogenstrukturen* could be observed. No macro-charcoal was found in the greyish siltstones.

### Macro-charcoal anatomy

Under SEM, the fragments show well-preserved anatomical details (Fig 3C) as well as homogenized cell walls (Fig 3D). It was possible to differentiate three anatomical patterns named as Porongos Charcoalified Wood Type 1, 2 and 3 (Table 1).

**Table 1:** Summary of the anatomical characteristics of the Porongos Charcoalified Wood Types 1, 2 and 3.

Wood tissue	Wood type 1	Wood type 2	Wood type 3
**Primary xylem**	Non-preserved	Non-preserved	Preserved
Tracheids width	-	-	19.9μm (average)
Pitting	-	-	Contiguous, scalariform
Pits	-	-	Narrow elongate, with 15.1μm (average) of width, and 1.9μm (average) high
Homogenised cell wall width	-	-	2.2μm
**Secondary xylem**	Pycnoxylic	Pycnoxylic	Pycnoxylic
Taxonomic affinity	*Agathoxylon* sp.	*Agathoxylon* sp.	*Agathoxylon* sp.
Axial parenchyma	Absent	Absent	Absent
Tracheids width	16.7μm (average)	20.1μm (average)	19.6μm (average)
Pitting	1–2 seriate, sub-oppositely to alternately arranged	1–4 seriate, alternately arranged	1–2 seriate, alternately arranged
Pits	Bordered, elliptical to narrow elongate elliptical	Bordered, circular to elliptical	Bordered, circular to elliptical
Ray type	Apparently homocelular	Homocelular	Homocelular
Ray width	Uniseriate	Non-preserved	Non-preserved
Ray height	2–7 cells	3–12 cells	2–4 cells
Ray cells	Apparently procumbent, 24.6μm (average) high	Procumbent, 22.7μm (average) high, and 75.9μm (average) in length.	Procumbent, 26.4μm (average) high, and 92.4μm (average) in length.
Cross-field pitting	Non-preserved	Araucarioid, 5–8 pits per field	Araucarioid, 5–8 pits per field
Homogenised cell wall width	2.2μm	1.9μm	1.9μm
Level of occurrence	2, 4, 5 and 6	1, 5 and 6	3 and 6

#### Porongos Charcoalified Wood Type 1 (S1 Fig)

Pycnoxylic secondary wood with 16.7 μm (11.3–21.1 μm) wide tracheids, showing 1–2 seriate, sub-oppositely to alternately arranged pitting. Pits are bordered and contiguously distributed, ranging in shape from elliptical with 6.4 μm (4.3–9.6 μm) in width and 5.2 μm (4.1–6.5 μm) in height, to narrow elongate elliptical with 7.1 μm (5.3–10.1 μm) in width and 2.6 μm (1.9–3.3 μm) in height. Apertures are elliptical and damaged by charring. Axial parenchyma absent. Rays are homocellular and uniseriate, 2–7 cells in height. Ray-cells are apparently procumbent and 24.6 μm (17.8–31.9 μm) in height, with non-measurable width due the fragmentation. Cell walls are homogenized with 2.2 μm (1.2–3.3 μm) in width. Cross-field pits are inconspicuous, and growth rings are not visible. This charcoalified wood type occurs in carbonaceous levels 2, 4, 5 and 6.

#### Porongos Charcoalified Wood Type 2 (S2 Fig)

Pycnoxylic secondary wood, tracheids 20.1 μm (12.1–33 μm) wide, exhibiting 1–4 seriate alternately arranged pitting. Pits are bordered and contiguous to semi-contiguous, ranging in shape from circular with 4.5 μm (3.3–6.5 μm) in diameter, to elliptical with 5.8 μm (4.1–6.7 μm) in width and 3.6 μm (2.9–4.5 μm) in height. Apertures are damaged by charring and are not clearly visible. Axial parenchyma absent. Rays with 3–12 cells in height and presence of radial parenchyma. Tangential view of rays not observed. Ray-cells are procumbent with 22.7 μm (17.5–29.9 μm) in height and 75.9 μm (50.3–98.5 μm) in width. Cross-field pitting is araucarioid, composed of 5–8 pits [10.8 μm (7.9–12.5 μm) in width and 7.6 μm (6.5–8.6 μm) in height] per field. Cell walls are homogenized with 1.9 μm (1.3–2.9μm) in width. Growth rings are not visible. This charcoalified wood type occurs in carbonaceous levels 1, 5 and 6.

#### Porongos Charcoalified Wood Type 3 (S3 Fig)

Wood exhibiting the transition between primary and secondary xylem. Primary xylem containing tracheids with 19.9 μm (14.4–23.5 μm) in width, showing contiguous narrow elongate scalariform pitting with 15.1 μm (8.2–22.6 μm) in width and 1.9 μm (11.1–3.9 μm) in height. Secondary wood is pycnoxylic and bears tracheids with 19.6 μm (13.5–26.4 μm) in width, exhibiting 1–2 alternately arranged seriate pitting. Pits are bordered, ranging in shape from circular with 7.2 μm (6.4–8.6 μm) in diameter, to elliptical with 8.1 μm (5.9–11.1 μm) in width and 5.6 μm (4.7–7 μm) in height. Apertures are damaged by charring and not clearly observable. Radial parenchyma. Tangential view of rays is not seen. Ray-cells are procumbent with 26.4 μm (21.9–30.7 μm) in height and 92.4 μm (79.4–105.7 μm) in length. Cross-field pitting is araucarioid and composed of 5–8 alternately arranged bordered pits [6.7 μm (4.1–8.7 μm) in width and 4.8 μm (3.6–5.5 μm) in height] per field. Cell walls are homogenized and 2.2 μm (1.2–3.2 μm) wide. Growth rings are not visible. This charcoalified wood type occurs in carbonaceous levels 3 and 6.

### Coal petrography

#### Carbonaceous Level 1

**Fig 4:**
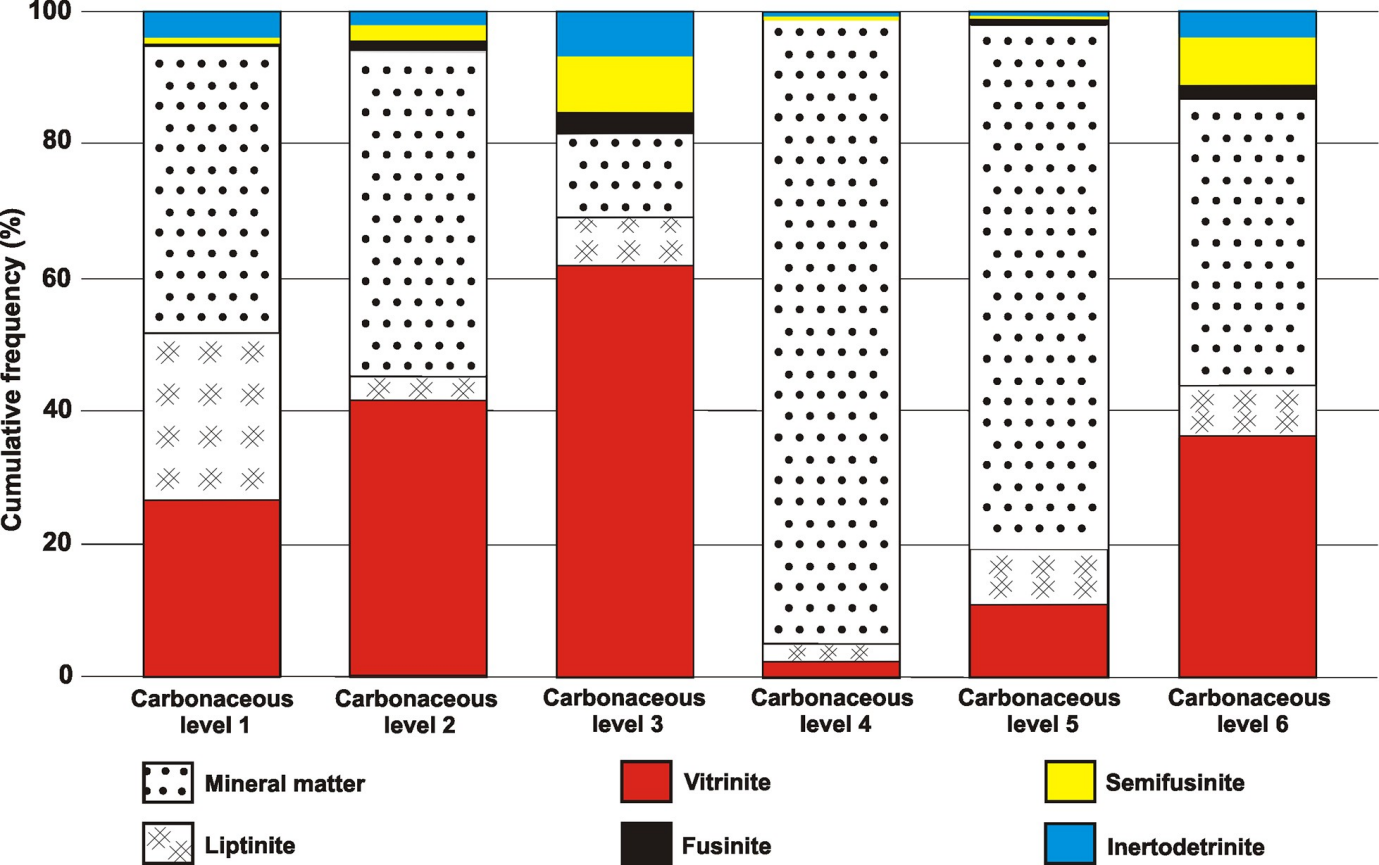
Maceral content of the six carbonaceous levels of the Barro Branco coal seam studied site. Values available on S1 Table.

Organic content (57%) is mainly composed of macerals of the vitrinite (26.8%) and liptinite (exclusively sporinite) groups (25%). The inertinite group is composed of fusinite (0.6%), semifusinite (0.8%) and inertodetrinite (3.8%). Mineral content (43%) is composed of clay (38.4%), pyrite (4.4%) and quartz (0.2%) (Fig 4). The reflectance value of vitrinite ranges from 0.619% to 0.86% (average 0.727%) and the reflectance value of inertinites ranges from 1.10% to 5.20% (average 2.00%) (Table 2).

**Table 2:** Reflectance values of vitrinites and inertinites of the six carbonaceous levels studied. Carbonaceous level (CL), Average (Avg), Standard deviation (SD), Minimum (Min), Maximum (Max) and number of measurements (n).

	Vitrinite reflectance (*%*)					Inertinite reflectance (*%*)					Charring temp. (^o^C)		
Level	Avg	SD	Min	Max	n	Avg	SD	Min	Max	n	Avg	Min	Max
CL 1	0.727	0.052	0.619	0.86	100	2.00	0.79	1.10	5.20	50	420	313	797
CL 2	0.723	0.069	0.558	0.853	100	2.40	1.05	0.90	6.60	50	467	290	962
CL 3	0.753	0.064	0.57	0.895	100	2.79	1.08	1.10	5.80	50	513	313	868
CL 4	0.692	0.006	0.509	0.827	100	1.88	0.60	0.90	3.40	50	405	290	585
CL 5	0.77	0.07	0.584	0.927	100	1.92	0.56	1.00	3.20	50	410	302	561
CL 6	0.791	0.073	0.626	0.982	100	2.58	1.37	0.90	7.30	50	488	290	1,045

#### Carbonaceous Level 2

Organic content (51%) is mainly composed of macerals of the vitrinite (41.4%) and liptinite (exclusively sporinite) groups (4.2%). The inertinite group is composed of fusinite (1.4%), semifusinite (2.2%) and inertodetrinite (2%). Mineral content (48.8%) is composed of clay (35.6%), pyrite (13%) and quartz (0.2%) (Fig 4). The reflectance values of vitrinite range from 0.558% to 0.853% (average 0.723%) and the reflectance values of inertinites range from 0.9% to 6.6% (average 2.40%) (Table 2).

#### Carbonaceous Level 3

Organic content (87%) comprises macerals of the vitrinite (61.8%) and liptinite (exclusively sporinite) groups (7.6%). The inertinite group is composed of fusinite (3%), semifusinite (8.2%) and inertodetrinite (7%). Mineral content (12.4%) is composed of clay (10%), pyrite (1.8%) and quartz (0.6%) (Fig 4). The reflectance values of vitrinite range from 0.57% to 0.895% (average 0.753%) and the reflectance values of inertinites range from 1.10% to 5.80% (average 2.79%) (Table 2).

#### Carbonaceous Level 4

Organic content (6%) consists macerals of the vitrinite (2.4%) and liptinite (exclusively sporinite) groups (2.6%). The inertinite group is composed of fusinite (0.6%), and inertodetrinite (0.4%). Mineral content (94%) is composed exclusively of clay (Fig 4). The reflectance values of vitrinite range from 0.509% to 0.827% (average 0.692%) and the reflectance values of inertinites range from 0.90% to 3.40% (average 1.88%) (Table 2).

#### Carbonaceous Level 5

Organic content (21%) contains macerals of the vitrinite (11.4%) and liptinite (exclusively sporinite) groups (8%). The inertinite group is composed of fusinite (0.6%), semifusinite (0.2%) and inertodetrinite (0.8%). Mineral content (79%) comprises clay (76.4%) and pyrite (2.6%) (Fig 4). The reflectance value of vitrinite ranges from 0.584% to 0.927% (average of 0.77%) and the reflectance values of inertinites ranges from 1.00% to 3.20% (average of 1.92%) (Table 2).

#### Carbonaceous Level 6

Organic content (57%) is mainly composed of macerals of the vitrinite (36.2%) and liptinite (exclusively sporinite) groups (7.8%). The inertinite group is composed of fusinite (2.2%), semifusinite (7.2%) and inertodetrinite (3.6%). Mineral content (43%) is composed of clay (40.6%) and pyrite (2.4%) (Fig 4). The reflectance values of vitrinite range from 0.626% to 0.982% (average 0.791%) and the reflectance values of inertinites range from 0.90% to 7.30% (average 2.58%) (Table 2).

## Discussion

### Palaeoenvironment and taphonomy

**Fig 5:**
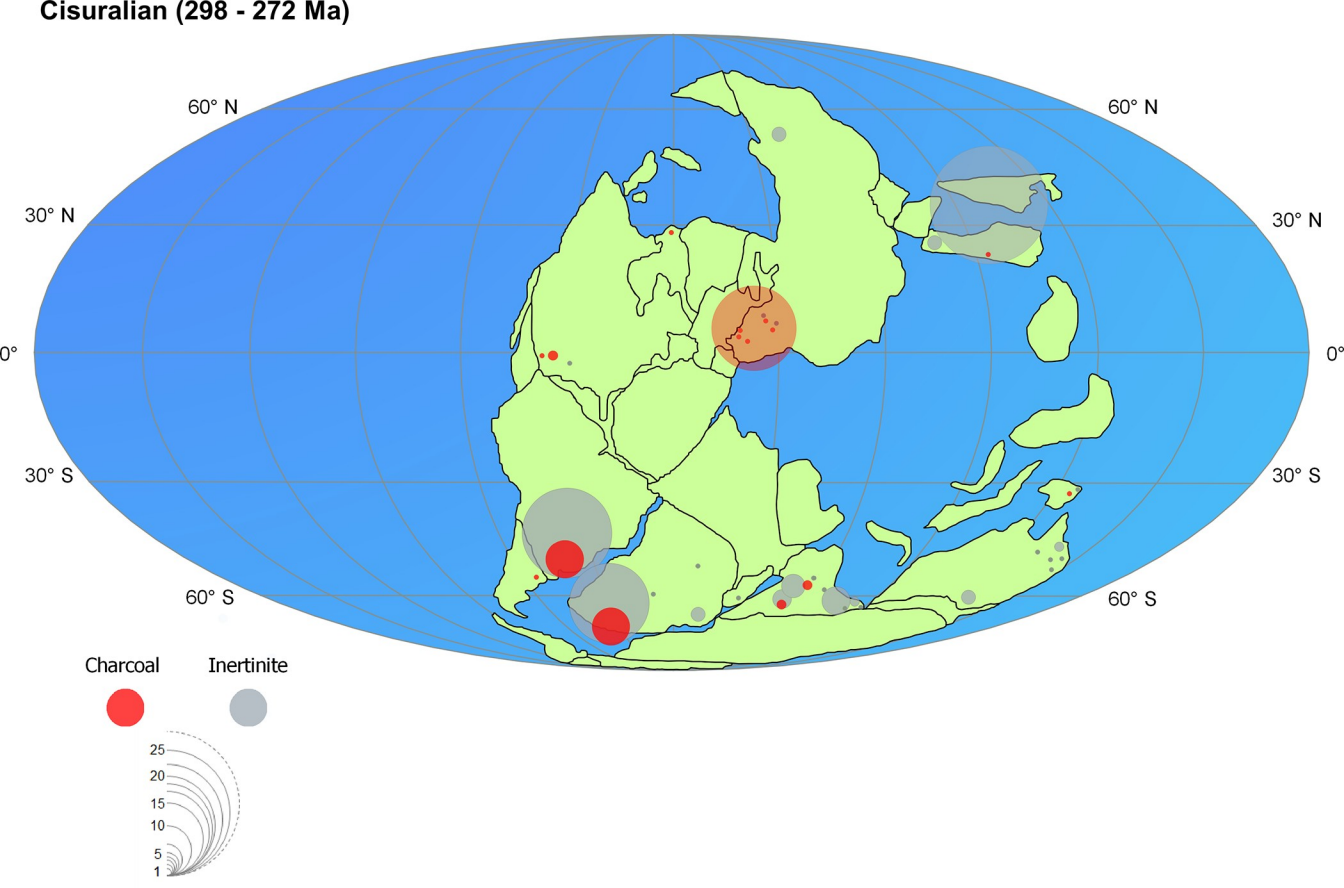
Global distribution of sedimentary charcoal and inertinites during the Cisuralian. Dots represent the number of described charcoal occurrences by basin and diameter varies according scale (detail of each occurrence in S2 Table and S3 Table). Map adapted from [35].

High inertinite contents in coals have frequently been reported from several Cisuralian coal deposits all over Gondwana (Fig 5) and in the Paraná Basin coal seams contents are variable, reaching >70% in some cases [1, 27, 28]. For the Barro Branco coal seam, a medium inertinite value of 14.6% was reported [15].

Macro-charcoal occurrences are also increasingly being reported for Gondwana (Fig 5), confirming that fire was a significant component of many terrestrial ecosystems on this palaeo-continent during the Cisuralian [4, 5, 6, 29]. In general, the Late Palaeozoic is considered a high-fire period of Earth’s history, as, due to considerably elevated atmospheric oxygen concentrations, the ignition and spread of wildfires were primarily controlled by atmospheric composition and not as during other periods by climate [2, 6, 30, 31, 32, 33, 34]. Under such conditions, fire events would have been frequent events even in ever-wet biomes such as mires [2, 3, 6, 33].

The co-occurrence between high-inertinite contents and macro-charcoal has been demonstrated in Gondwanan coal-bearing strata [4, 5], but analysis of both types of data for the same samples are still rare. Examples of such analysis come from the Cisuralian deposits of the Bonito mine I (Bonito coal seam, Rio Bonito Formation, Santa Catarina State, Brazil) [36] and Dhanpuri Coal Mine (Barakar Formation, Sohagpur Coalfield, Madhya Pradesh, India) [6].

Although a pyrogenic origin of fossil charcoal, or fusain, in clastic sediments as well as in coals and lignites has been widely accepted for the northern continents [37, 38, 39], the origin of the high-inertinite contents in the Permian Gondwanan coal-bearing strata remained an unresolved matter of debate amongst many coal-petrologists and palaeobotanists. Using integrated methods, it was demonstrated that the medium content of 42.2% inertinites in coals from the Dhanpuri Coal Mine in India was coincident with the occurrence of macro-charcoal, indicating that palaeo-wildfires reached the mire during the deposition of this peat [6]. The occurrence of macro-charcoal in the six carbonaceous levels of the Porongos outcrop indicates that fire was a recurrent element in the depositional system studied here. However, inertinite contents vary from 1.0% in Carbonaceous Level 4 up to 18.2% in Carbonaceous Level 3 (= 7.4% medium).

Modern peat-forming environments are susceptible to surface or smouldering ground wildfires, especially during seasons of severe drought or during longer periods of reduced water tables [40, 41, 42, 43, 44]. Under these conditions, surficial burning of previously deposited peat layers can be represented by a continuous layer of charred material [45]. Such surface fires might ignite smouldering ground fires [46] and produce large quantities of charred remains [17, 18]. Such continuous bands, rich in charred material, including abundant macro-charcoal and inertinites have not been found in any of the six carbonaceous levels investigated here. Therefore, in the studied area, there is no clear evidence to support an autochthonous surface or a smouldering ground burning of peat-forming material inside the mire.

The slightly abrasions observed on the edges of most macro-charcoal recovered from the six carbonaceous levels might indicate that such charred material was transported into the mire. This suggests that the fire events occurred some distance away from the place of final deposition. As the size of the macro-charcoal remains investigated here ranges from fragments of 3 mm x 5 mm to relatively larger fragments of 49 mm x 111 mm, they were transported inside the depositional environment via hydraulic flow and not by wind [18, 47]. That water transport may have resulted in a selective bias in favour of macroscopic charred wood remains, as no other charred plant organs were recorded in the six carbonaceous levels studied here [17, 48]. This taphonomical interpretation is congruent with the high mineral content present in all six carbonaceous levels, which suggest that the water influxes frequently transported sediments from an external source into the mire. Although macro-charcoal was transported by water and wind might also have acted and transported the minor charcoal particles into or within the mire. Such particles are petrographically represented by inertodetrinites, which are present in all the six carbonaceous levels. These fine charcoal particles can be lifted into the air and transported over the long distances, and might be formed by crown fires [17, 49, 50].

A reliable determination of the transport distance of the charred remains studied here is difficult. In modern environments, the presence of macro-charcoal in forest soils as well as lake and peat-forming deposits is usually considered as an indicator of a local wildfire event [51, 52, 53, 54, 55, 56, 57]. As the fossil assemblages studied here are composed predominantly of macro-charcoal remains (some of them of relatively large size), a hypautochthonous origin for such charred material might be suggested. This interpretation may be supported by the presence of only slight abrasions on the edges of the macro-charcoal from all six carbonaceous levels, which suggest that these fossils were transported only over the short distances [58]. Therefore, it seems that these recurrent palaeo-wildfires events occurred in the vicinity of the Barro Branco coal seam peat-forming environment. The lack of charred remains in the massive shale levels might be the result of an absence of palaeo-wildfires during the deposition of these sedimentary horizons or it might be a result of a taphonomical bias, since no other plant fossil remains where documented in these sedimentary layers. However, it is well-know that charcoal can be preserved in non-carbonaceous silty-grained sediments [18, 58].

### Charring temperature and palaeo-wildfire classification

Experimental studies demonstrated that the reflectance values of charred plant tissues increase with increasing of charring temperatures [18, 59, 60, 61, 62]. Therefore, reflectance values of charcoal and inertinites have been used to estimate palaeo-wildfire temperatures [3, 6, 60, 63, 64, 65]. The average of the inertinite reflectance values from the six carbonaceous levels studied ranged from 1.88 (%) to 2.79 (%), and this indicates an estimated medium charring temperature that range from 405 to 513°C for these recurrent palaeo-wildfires (Table 2). As the average of the vitrinite reflectance values from the six carbonaceous levels are relatively low, ranging from 0,692 (%) to 0,791 (%) (Table 2), coalification did not affect inertinite reflectance [63]. However, the maximum reflectance value of 7.3%, indicates a charring temperature of 1,045°C.

Traditionally, wildfires have been classified into surface, ground and crown fires [17, 46, 66, 67]. Surface fire burns dead plant material derived from litter as well as living shrubby and herbaceous plants, and have a comparably low temperature [17, 46, 66, 68]. This type of fire produce most of the macro-charcoal [18], and might ignite smouldering ground fires, which burn organic-rich soil layers beneath the surface litter at low temperatures [17, 46, 69] and can last from days to years [70]. In contrast, crown fires have high temperatures, burn living vegetation from canopy as well as understorey trees [17, 46, 66, 71], and produce less macro-charcoal [18].

As the charred remains from the six carbonaceous levels studied here were transported, an *in-situ* observation of the depositional characteristics of these repeated wildfire events is not possible. Therefore, it is difficult to classify the fire type that produced the charred materials as well as indicate the fire intensity. However, maximum burning temperatures reached up to 1,045°C and suggest that at least in some cases intensive heat acted on the wood. Based on the predominance and relatively large size of the macro-charcoal remains in the six carbonaceous levels studied here, we speculate that they are result of repetitive surface palaeo-wildfires, as such a fire type produce most of the macro-charcoal [18, 72]. However, high-temperature crown fires might have also played a (subordinate) role in forming the macro-charcoal within the six carbonaceous levels.

Considering the taphonomical interpretation presented above, these charred remains produced by such palaeo-wildfires were transported and hydrodynamically sorted only over a short distance into the mire. This might explain the low abundance of macro-charcoal and inertinites in the six carbonaceous levels studied here, as not all charred remains produced by these fires may have reached the mire. It has been suggested that much of the inertinites present in Permian Gondwana coals resulted from low temperature surface palaeo-wildfires [17]. Some of these palaeo-wildfires occurred outside the mire and may have been followed by increased soil erosion, which could explain the high mineral content present not only in the coal seams analysed here, but also in many other Permian Gondwana coal deposits [17].

### Taxonomical affinities and palaeoecological considerations

Although the charcoalified remains exhibit exceptional well-preserved internal anatomical details, due to their fragmentation and possible alterations at the time of charring, it is possible to establish a general taxonomic affinity for them. In charcoalified wood types 1 and 2 only the secondary xylem is preserved. In contrast, charcoalified wood type 3 exhibits part of the primary xylem, which is rarely preserved in Late Palaeozoic Gondwana woods [73]. This preserved part is small, and therefore, it seems more appropriate to establish the taxonomic affinity of charcoalified wood type 3 on the basis of secondary xylem, as more anatomical features such as tracheid pitting, rays and cross-field pitting are preserved in this tissue.

The three wood types identified here are rather similar in their secondary xylem characteristics and exhibit a typical gymnospermous anatomical pattern. The major anatomical differences between the secondary xylem of charcoalified wood types identified here, is that wood type 1 has tracheids with 1–2 seriate pitting and rays which are 2–7 cells high, while wood type 2 has tracheids with 1–4 seriate pitting and rays which are 3–12 cells high and wood type 3 has rays with 2–4 cells high (Table 1). These charcoalified wood types may not represent natural taxa, and it is quite possible that they are different morphological stages of distinct parts or ontogenetic stages of the same taxon [74]

The presence of tracheids bearing contiguous uniseriate and alternate multiseriate bordered pitting and the absence of axial parenchyma, allows for a generic classification of the secondary xylem of three wood types as *Agathoxylon* Hartig [75, 76, 77]. This taxonomical definition is more reliable for wood type 2 and 3 as both have araucarioid cross-field pitting, which is a typical anatomical pattern of *Agathoxylon* fossil wood secondary xylem [75, 76, 77]. This wood anatomical pattern is widespread in Late Palaeozoic and Mesozoic deposits of different geographical regions of the both hemispheres [5, 73, 77, 78, 79, 80]. It is related to several gymnosperm groups such as Cycadales, Caytoniales, Glossopteridales, Cordaitales, Voltziales, Ullmanniales, Cheirolepidiaceae and Araucariaceae [81, 82].

Macro-charcoal remains presenting secondary xylem with an *Agathoxylon* anatomical pattern have already been reported from several Lower Permian coal-bearing deposits of the Rio Bonito Formation [4, 7, 8, 83, 84]. In the Faxinal coalfield (a locality of the Rio Bonito Formation), macro-charcoal remains with such an anatomy occur in association with abundant *Glossopteris* leaves, suggesting a biological connection between both [8, 84]. However, until now there is no unequivocal evidence, which could definitively indicate that to which group or groups of gymnosperms these *Agathoxylon*-like charcoals from the Rio Bonito Formation might really belong.

As the charcoalified assemblages of the six carbonaceous levels studied here are composed exclusively of the *Agathoxylon* type of wood, it is possible to infer that plants possessing such an anatomical pattern were the most important components of the biomass responsible for the maintenance of these repetitive palaeo-wildfires. The uniformity of the vegetation in the charcoalified assemblages of the six carbonaceous levels might suggest that these plants were well adapted to grow in an environmental, experiencing the repeated and recurrent palaeo-wildfires events.

Fire-adaptation is an ecological-evolutionary trend, observed in modern environment vegetation that is submitted to regular wildfires [85, 86, 87, 88], and has been considered as a key-factor in the evolution of Palaeozoic early conifers [89]. In the Faxinal coalfield, which can be considered as a palaeoenvironment disturbed by palaeo-wildfires and volcanic activity [8, 90], the presence of an abaxial trichome complex on *Glossopteris pubescens* leaves, a general xeromorphic feature of leaves in a wide variety of taxa, has been mentioned as a possible mechanism providing insulation against the heat produced by fire [91]. However, until now there is no clear evidence of any fire-adapted morphological structure in wood remains from Rio Bonito Formation, and further discoveries of fossil wood might shed some light about this complex ecological-evolutionary relation.

## Conclusions

Based on the data and interpretations presented here, the following conclusions regarding the charcoalified assemblages from the six Barro Branco coal seams can be drawn:

1. The presence of macro-charcoal as well as inertinites in all six carbonaceous levels, provide the first evidence of the occurrence of repeated palaeo-wildfires during the deposition of the Barro Branco coal seam.

2. These recurrent palaeo-wildfires are related to the high palaeo-atmospheric O_2_ concentrations proposed for the Lower Permian.

3. The macro-charcoal remains were transported inside the mire depositional system via hydraulic flow, and such water transport may have resulted in a selective bias in favour of charred wood remains. However, wind blow could also have acted and transported minor charcoal particles (inertodetrinites) inside the mire.

4. A hypoautochtonous origin for the macro-charcoal of all six levels might be suggested, and therefore, these repeated palaeo-wildfires events occurred in the vicinity of the Barro Branco coal seam peat-forming.

5. The reflectance values from the inertinites of all six levels indicate an estimated charring temperature reaching up to 1,045°C. This high charring temperature represents intense fire acting in the depositional system.

6. The study of the macro-charcoal from the six carbonaceous levels indicates the presence of secondary xylem with an *Agathoxylon*-like anatomical pattern.

7. The non-significant variation of the wood anatomy in the charcoalified assemblages throughout the six levels suggest that gymnospermous plants bearing *Agathoxylon*-like secondary xylem were one of the most important components of the biomass responsible for the maintenance of these recurrent palaeo-wildfires.

## Supporting information

S1 Fig Anatomical details of Porongos Charcoalified Wood Type 1. A, B and C) Tracheids exhibiting 1–2 seriate sub-oppositely to alternately arranged pitting. B and C) Bordered pits with an elliptical to narrow elongate elliptical shape. B) Apertures damaged by charring process, however apparently elliptical. D) Uniseriate rays with 2–7 cells in height. E) Homocellular rays bearing apparently procumbent cells. F) Homogenized cell walls. All fragments were extracted from rock sample PBUMCN 1168.
(TIF)

S2 Fig Anatomical details of Porongos Charcoalified Wood Type 2. A and B) Tracheids with 1–4 seriate alternately arranged pitting; pits are bordered ranging in shape from circular to elliptical. C and D) Rays with 3–12 cells in height and presence of radial parenchyma. E) Procumbent ray cells with araucarioid cross-field pitting composed of 5–8 pits per field. F) Homogenized cell walls. Fragments (A, B, D, E and F) extracted from PBUMCN 1163, and fragment (C) extracted from PBUMCN 1167.
(TIF)

S3 Fig Anatomical details of Porongos Charcoalified Wood Type 3. A) Transition between primary and secondary xylem. B) Primary xylem tracheids exhibiting contiguous narrow elongate scalariform pitting, and homogenized cell walls. C) Secondary xylem tracheids exhibiting 1–2 alternately arranged seriate pitting; pits ranging in shape from circular to elliptical. D) Rays with procumbent cells and presence of radial parenchyma. E) Araucarioid cross-field pitting composed of 5–8 alternately arranged bordered pits per field. F) Secondary xylem homogenized cell walls. Fragments (A, C, D, E and F) extracted from rock sample PBUMCN 1165, and fragment (B) extracted from sample PBUMCN 1168.
(TIF)

S1 Table Maceral content of the six carbonaceous levels of the Barro Branco coal seam studied site. (DOCX)

S2 Table Published records of charcoal in Lower Permian. Data based on [1,2,3,4], and additional sources not mentioned in these previous compilations.
(DOCX)

S3 Table Published records of inertinites in Lower Permian coals. Data based on [1,2,3,4,5], and additional sources not mentioned in these previous compilations.
(DOCX)
