# Supplementary figures and images for: Recurrent palaeo-wildfires in a Cisuralian coal seam: A palaeobotanical view on high-inertinite coals from the Lower Permian of the Paraná Basin, Brazil
Source: PLoS One. 2019 Mar 14;14(3):e0213854. doi: 10.1371/journal.pone.0213854 (PMC6417680; doi:10.1371/journal.pone.0213854)

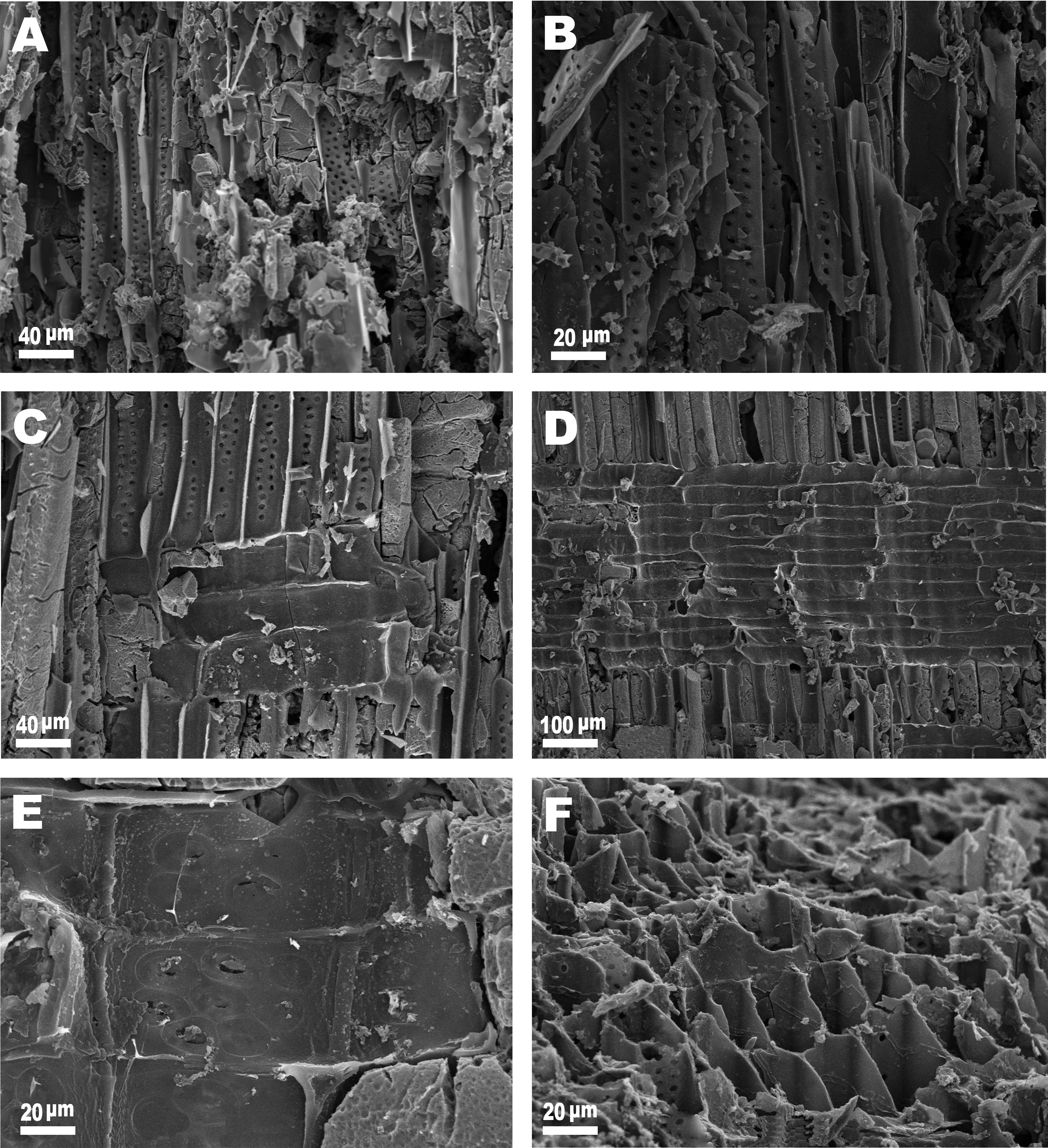

Supplement: S2 Fig — A and B) Tracheids with 1–4 seriate alternately arranged pitting; pits are bordered ranging in shape from circular to elliptical. C and D) Rays with 3–12 cells in height and presence of radial parenchyma. E) Procumbent ray cells with araucarioid cross-field pitting composed of 5–8 pits per field. F) Homogenized cell walls. Fragments (A, B, D, E and F) extracted from PBUMCN 1163, and fragment (C) extracted from PBUMCN 1167. (TIF) [file pone.0213854.s002.tif]

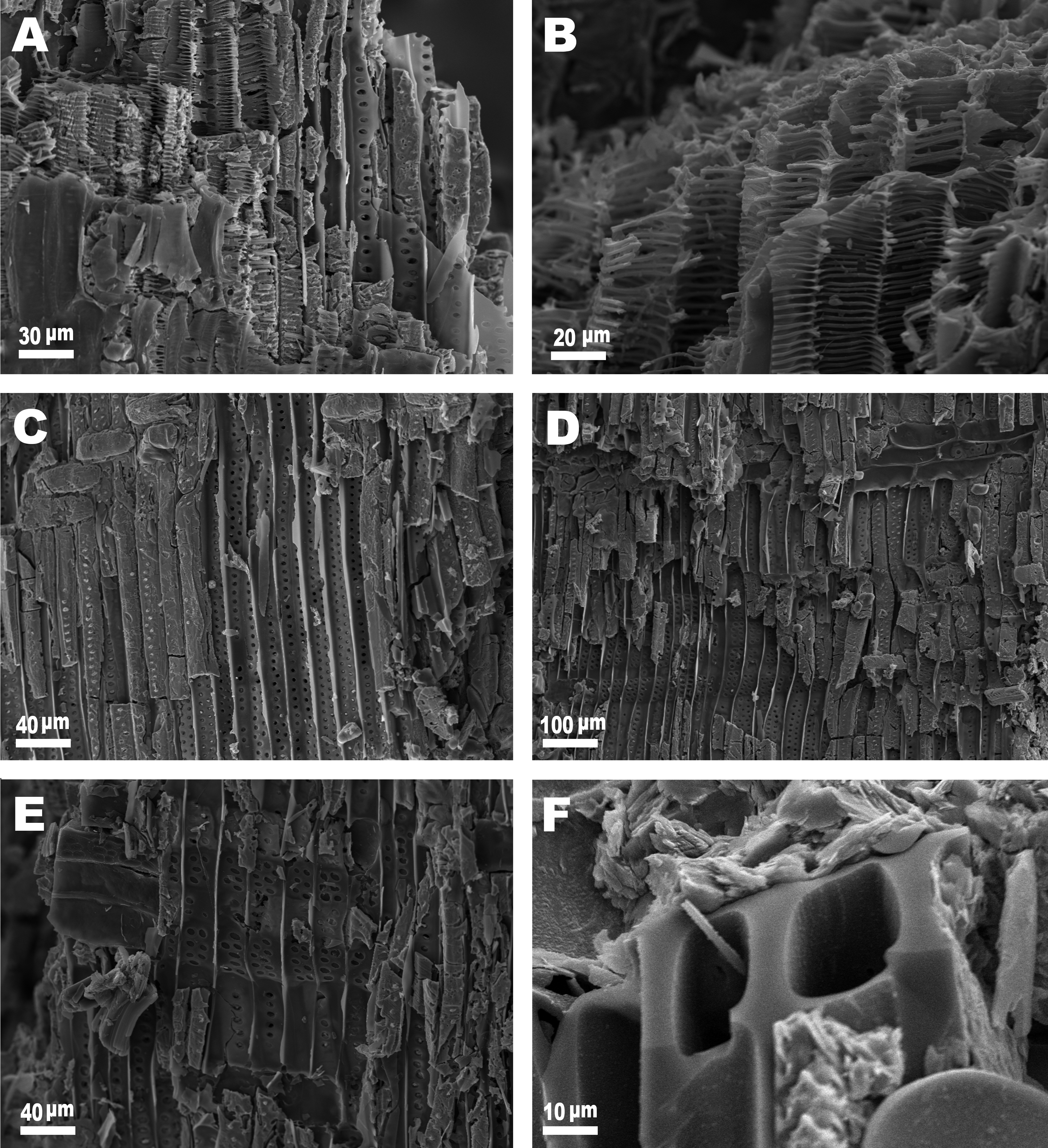

Supplement: S3 Fig — A) Transition between primary and secondary xylem. B) Primary xylem tracheids exhibiting contiguous narrow elongate scalariform pitting, and homogenized cell walls. C) Secondary xylem tracheids exhibiting 1–2 alternately arranged seriate pitting; pits ranging in shape from circular to elliptical. D) Rays with procumbent cells and presence of radial parenchyma. E) Araucarioid cross-field pitting composed of 5–8 alternately arranged bordered pits per field. F) Secondary xylem homogenized cell walls. Fragments (A, C, D, E and F) extracted from rock sample PBUMCN 1165, and fragment (B) extracted from sample PBUMCN 1168. (TIF) [file pone.0213854.s003.tif]
