# Supplementary material for: Recurrent palaeo-wildfires in a Cisuralian coal seam: A palaeobotanical view on high-inertinite coals from the Lower Permian of the Paraná Basin, Brazil
Source: PLoS One. 2019 Mar 14;14(3):e0213854. doi: 10.1371/journal.pone.0213854 (PMC6417680; doi:10.1371/journal.pone.0213854)
Supplement: S1 Table — (DOCX) [file pone.0213854.s004.docx]

**S1 Table.** Maceral content of the six carbonaceous levels of the Barro Branco coal seam studied site.

|  | **Level 1** | **Level 2** | **Level 3** | **Level 4** | **Level 5** | **Level 6** |
| --- | --- | --- | --- | --- | --- | --- |
| **Total Vitrinite (vol.%)** | **26.8** | **41.4** | **61.8** | **2.4** | **11.4** | **36.2** |
| Cutinite | 0 | 0 | 0 | 0 | 0 | 0 |
| Sporinite | 25 | 4.2 | 7.6 | 2.6 | 8 | 7.8 |
| Liptodetrinite | 0 | 0 | 0 | 0 | 0 | 0 |
| **Liptinite total (vol.%)** | **25** | **4.2** | **7.6** | **2.6** | **8** | **7.8** |
| Fusinite | 0.6 | 1.4 | 3 | 0 | 0.6 | 2.2 |
| Semifusinite | 0.8 | 2.2 | 8.2 | 0.6 | 0.2 | 7.2 |
| Inertodetrinite | 3.8 | 2 | 7 | 0.4 | 0.8 | 3.6 |
| **Inertinite (vol.%)** | **5.2** | **5.6** | **18.2** | **1** | **1.6** | **13** |
| Clay | 38.4 | 35.6 | 10 | 94 | 76.4 | 40.6 |
| Carbonate | 0 | 0 | 0 | 0 | 0 | 0 |
| Quartz | 0.2 | 0.2 | 0.6 | 0 | 0 | 0 |
| Pyrite | 4.4 | 13 | 1.8 | 0 | 2.6 | 2.4 |
| **Mineral matter (vol.%)** | **43** | **48.8** | **12.4** | **94** | **79** | **43** |
| **Organic matter (vol.%)** | **57** | **51.2** | **87.6** | **6** | **21** | **57** |
| **Total (vol.%)** | **100** | **100** | **100** | **100** | **100** | **100** |
