# Supplementary material for: Recurrent palaeo-wildfires in a Cisuralian coal seam: A palaeobotanical view on high-inertinite coals from the Lower Permian of the Paraná Basin, Brazil
Source: PLoS One. 2019 Mar 14;14(3):e0213854. doi: 10.1371/journal.pone.0213854 (PMC6417680; doi:10.1371/journal.pone.0213854)
Supplement: S2 Table — Data based on [1,2,3,4], and additional sources not mentioned in these previous compilations. (DOCX) [file pone.0213854.s005.docx]

**S2 Table**. Published records of charcoal in Lower Permian. Data based on [1,2,3,4], and additional sources not mentioned in these previous compilations.

| Locality | Basin | Country | Age | Evidence type | References |
| --- | --- | --- | --- | --- | --- |
|  |  |  |  |  |  |
| Manebach | Thuringian Forest Basin | Germany | Asselian | Macro-charcoal | [5,6,7] |
| Oberhausen/Appel | Saar-Nahe Basin | Germany | Asselian | Macro-charcoal | [8] |
| Rehborn | Saar-Nahe Basin | Germany | Asselian | Macro-charcoal | [8] |
| Kreimbach, Kaulbach | Saar-Nahe Basin | Germany | Asselian | Macro-charcoal | [8,9] |
| Challodenbach | Saar-Nahe Basin | Germany | Asselian | Macro-charcoal | [8] |
| Kahleckerhof | Saar-Nahe Basin | Germany | Asselian | Macro-charcoal | [8] |
| Wingertsweilerhof | Saar-Nahe Basin | Germany | Asselian | Macro-charcoal | [8] |
| Heiligenmoschel | Saar-Nahe Basin | Germany | Asselian | Macro-charcoal | [8] |
| Obermoschel | Saar-Nahe Basin | Germany | Asselian | Macro-charcoal | [10] |
| Alsenz | Saar-Nahe Basin | Germany | Asselian | Macro-charcoal | Uhl, unpubl. data |
| Niedermoschel | Saar-Nahe Basin | Germany | Asselian | Macro-charcoal | Uhl, unpubl. data |
| Meisenheim | Saar-Nahe Basin | Germany | Asselian | Macro-charcoal | Uhl, unpubl. data |
| Grumbach | Saar-Nahe Basin | Germany | Asselian | Macro-charcoal | Uhl, unpubl. data |
| Jambi, Mengkarang Formation | - - - - - | Indonesia | Asselian- Sakmarian | Macro-charcoal | [11] |
| Testemunho PANG0001 (província de Buenos Aires) | Claromecó Basin | Argentina | Asselian- Sakmarian | Macro-charcoal | [12] |
| Schallodenbach | Saar-Nahe Basin | Germany | Sakmarian | Macro-charcoal | [8] |
| Kahleckerhof | Saar-Nahe Basin | Germany | Sakmarian | Macro-charcoal | [8] |
| Wingertsweilerhof | Saar-Nahe Basin | Germany | Sakmarian | Macro-charcoal | [8] |
| Heiligenmoschel | Saar-Nahe Basin | Germany | Sakmarian | Macro-charcoal | [8] |
| Hirschhorn | Saar-Nahe Basin | Germany | Sakmarian | Macro-charcoal | Uhl et al., in prep. |
| Olsbrücken | Saar-Nahe Basin | Germany | Sakmarian | Macro-charcoal | Uhl et al., in prep. |
| Chemnitz | Chemnitz Basin | Germany | Sakmarian | Macro-charcoal | [13] |
| Geraldine Bonebed, Archer Country, Texas | Midland Basin | United States | Sakmarian | Macro-charcoal | [14,15] |
|  |  |  |  |  |  |

(continued)

| Locality | Basin | Country | Age | Evidence type | References |
| --- | --- | --- | --- | --- | --- |
| Rattlesnake Canyon 2,  Archer Country, Texas | Midland Basin | United States | Sakmarian | Macro-charcoal | [16] |
| Doña Ana County, New Mexico | Orogrande Basin | United States | Sakmarian | Macro-charcoal | [17,18] |
| Morro do Papaléo mine | Paraná Basin | Brazil | Sakmarian | Macro-charcoal | [19,20] |
| Candiota coalfield | Paraná Basin | Brazil | Sakmarian | Macro-charcoal | [20] |
| Leão Butiá coal-field | Paraná Basin | Brazil | Sakmarian | Macro-charcoal | [20] |
| Faxinal coalfield | Paraná Basin | Brazil | Sakmarian | Macro-charcoal | [20,21,22,23,24,25] |
| Quitéria outcrop | Paraná Basin | Brazil | Sakmarian | Macro-charcoal | [26,27,28,29,30,31] |
| Bonito coal mine | Paraná Basin | Brazil | Sakmarian- Artinskian | Macro-charcoal | [32,33-20] |
| Figueira coalfield | Paraná Basin | Brazil | Artinskian | Macro-charcoal | [20] |
| Porongos outcrop | Paraná Basin | Brazil | Artinskian | Macro-charcoal | This work |
| Vereeniging coalfield | Karoo Basin | South Africa | Artinskian | Macro-charcoal | [2] |
| Ogies region | Karoo Basin | South Africa | Artinskian | Macro-charcoal | [34] |
| Spitzkop mine | Karoo Basin | South Africa | Artinskian | Macro-charcoal | [35] |
| Middelburg mine | Karoo Basin | South Africa | Artinskian | Macro-charcoal | [35] |
| Rietspruit mine | Karoo Basin | South Africa | Artinskian | Macro-charcoal | [35] |
| Goedehoop mine | Karoo Basin | South Africa | Artinskian | Macro-charcoal | [35] |
| Kromdraai mine | Karoo Basin | South Africa | Artinskian | Macro-charcoal | [35] |
| Kleinkopje mine | Karoo Basin | South Africa | Artinskian | Macro-charcoal | [35] |
| South Karanpura | South Rewa Gondwana Basin | India | Artinskian | Macro-charcoal | [36] |
| Danphuri coal mine | South Rewa Gondwana Basin | India | Artinskian | Macro-charcoal | [37] |
| Jawahar Khani-5 (Yellandu) | Goodavari Basin | India | Lower Permian | Microscopic | [38] |
| Chintalapudi | Goodavari  Basin | India | Lower Permian | Microscopic | [39] |
|  |  |  |  |  |  |
| Autanian | Autun Basin | France | Lower Permian | Macro-charcoal | [40] |
| Mont Sénégra | Lodève Basin | France | Lower Permian | Microscopic | [41] |
| Qiaotou (Baode) | Ordos Basin | China | Artinskian - Kungurian | Macro-charcoal | [4] |
| Axel Heiberg Island | Sverdrup Basin | Canada | Kungurian | ? | [42] |

**References**

1. Abu Hamad, A.M.A., Jasper, A., Uhl, D., 2012. The record of Triassic charcoal and other evidencefor palaeo-wildfires: signal for atmospheric oxygen levels, taphonomic biases or lack of fuel. International Journal of Coal Geology, 96, 60–71.
2. Jasper, A., Guerra-Sommer, M., Hamad, A., Abdalla, M.B., Bamford, M., Bernardes-de-Oliveira, M.E.C., Tewari, R., Uhl, D., 2013. The burning of Gondwana: permian fires on the southern continent: a palaeobotanical approach. Gondwana Research, 24(1), 148-160.
3. Jasper, A., Uhl, D., Tewari, R., Guerra-Sommer, M., Spiekermann, R., Manfroi, J., ... & da Rosa, Á. A. S. (2016). Incêndios vegetacionais Indo-Brasileiros no Neopaleozoico: uma revisão dos registros de carvão vegetal macroscópico. *Geologia USP. Série Científica*, *16*(4), 87-97.
4. Yan, M., Wan, M., He, X., Hou, X., Wang, J., 2016. First report of Cisuralian (Early Permian) charcoal layers within a coal bed from Baode, North China with reference to global wildfire distribution. Palaeogeography, Palaeoclimatology, Palaeoecology, 459, 394–408.
5. Remy, W., 1954. Laubfusit, ein Beitrag zur Frage der Fusitbildung. Berg. Z. Gluk, 90, 64–67.
6. Barthel, M., Rößler, R., 1997. Tiefschwarze Kieselstämme aus Manebach. Veröffentlichungen des Naturhistorischen Museums Schleusingen, 12, 53–61.
7. Barthel, M., 2008. Die Rotliegendflora des Thüringer Waldes Teil 6: Wurzeln und fertile Organe. Algen und Bakterien. Vegetation. Veröffentlichungen des Naturhistorischen Museums Schleusingen, 23, 41–67.
8. Uhl, D., Lausberg, S., Noll, R., Stapf, K., 2004. Wildfires in the Late Palaeozoic of Central Europe–an overview of the Rotliegend (Upper Carboniferous–Lower Permian) of the Saar–Nahe Basin (SW-Germany). Palaeogeography, Palaeoclimatology, Palaeoecology, 207, 23–35.
9. Schinder, T., Uhl, D., Noll, R., Bach, T., Hohn, W., Poschmann, M., Rahm, B., Schweiss, D., Wttike, M., 2004. Erstfunde von Sigillaria-Stämmen in situ in Rotliegend-Ablagerungen (Ober-Karbon bis Unter-Perm) der Nordpfalz (Südwestdeutschland). Neues Jahrbuch für Geologie & Paläontologie, Abhandlungen, 233(1), 1–26. Stuttgart.
10. Schindler, T., Poschmann, M., Schoch, R.R., Uhl, D., Voigt, S., 2014. Early Permian amphibians from the construction pit of a German wind power station: ecological and biogeographical inferences. Permophiles, 59, 20–23.
11. Crippa, G., Angiolini, L., Van Waveren, I., Crow, M., Hasibuan, F., Stephenson, M., Ueno, K., 2014. Brachiopods, fusulines and palynomorphs of the Mengkarang Formation (Early Permian, Sumatra) and their palaeobiogeographical significance. Journal of Asian Earth Sciences, 79, 206–223.
12. Arzadún, G., Cisternas, M.E., Cesaretti, N.N., Tomezzoli, R.N., 2017. Presence of charcoal as evidence of paleofires in the Claromecó Basin, Permian of Gondwana, Argentina: Diagenetic and paleoenvironment analysis based on coal petrography studies. GeoResJ, 14, 121–134.
13. Rößler, R., 2001. Der Versteinerte Wald von Chemnitz. Museum für Naturkunde, Chemnitz, Germany.
14. Sander, P.M., 1987. Taphonomy of the Lower Permian Geraldine bonebed in Archer County, Texas. Palaeogeography, Palaeoclimatology, Palaeoecology, 61, 221–236.
15. Sander, P.M., Gee, C.T., 1990. Fossil charcoal: techniques and applications. Review of Palaeobotany and Palynology, 63, 269–279.
16. Sander, P.M., 1989. Early Permian depositional environments and pond bonebeds in central Archer County, Texas. Palaeogeography, Palaeoclimatology, Palaeoecology, 69, 1–21.
17. Mack, G.H., Leeder, M., Perez-Arlucea, M., Bailey, B.D., 2003. Sedimentology, paleontology, and sequence stratigraphy of Early Permian estuarine deposits, south-central New Mexico, USA. Palaios, 18, 403–420.
18. Falcon-Lang, H.J., Kurzawe, F., Lucas, S.G., 2014. Coniferopsid tree trunks preserved in sabkha facies in the Permian (Sakmarian) Community Pit Formation in southcentral New Mexico, USA: systematics and palaeoecology. Review of Palaeobotany and Palynology, 200, 138–160.
19. Jasper, A., Manfroi, J., Schmidt, E.O., Machado, N.T.G., Konrad, O., Uhl, D., 2011a. Evidências paleobotânicas de incêndios vegetacionais no afloramento Morro do Papaléo, Paleozoico Superior do Rio grande do Sul, Brasil. Geonomos 19(1), 18–27.
20. Jasper, A., Uhl, D., Guerra-Sommer, M., Bernardes-de-Oliveira, M.E.C., Machado, N.T.G., 2011b. Upper Paleozoic charcoal remains from South America: multiple evidences of fire events in the coal bearing strata of the Paraná Basin, Brazil. Palaeogeography, Palaeoclimatology, Palaeoecology, 306(3–4), 205–218.
21. Jasper, A., Uhl, D., Guerra-Sommer, M., Abu Hamad, A., Machado, N.T.G., 2011c. Charcoal remains from a tonstein layer in the Faxinal Coalfield, Lower Permian, southern Paraná Basin, Brazil. Anais da Academia Brasileira de Ciências, 83(2), 471–481.
22. Guerra-Sommer, M., Cazzulo-Klepzig, M., Santos, J.O.S., Hartmann, L.A., Ketzer, J.M., Formoso, M.L.L., 2008b. Radiometric age determination of tonstein and stratigraphic constrains for the Lower Permian coal succession in southern Paraná Basin, Brazil. International Journal of Coal Geology, 74, 13–27.
23. Simas, M.W., Guerra-Sommer, M., Mendonça Filho, J.G., Cazzulo-Klepzig, M., Formoso, M.L.L., Degani-Schmidt, I., 2013. An accurate record of volcanic ash fall deposition as characterized by dispersed organic matter in a lower Permian tonstein layer (Faxinal Coalfield, Paraná Basin, Brazil). Geologica Acta: an international earth science jornal, 11(1), 45–47.
24. Degani-Schmidt, I., Guerra-Sommer, M., Oliveira Mendonça, J., Mendonça Filho, J.G., Jasper, A., Cazzulo-Klepzig, M., Iannuzzi, R., 2015. Charcoalified logs as evidence of hypautochthonous/autochthonous wildfire events in a peat-forming environment from the Permian of southern Paraná Basin (Brazil). International Journal of Coal Geology, 146, 55–67.
25. Degani-Schmidt, I., Guerra-Sommer, M., Freitas, T., 2018. Preserved Cytoplasm in Charred *Agathoxylon*-type Wood from the Permian of Brazilian Paraná Basin. Revista Brasileira de Paleontologia, 21(2), 112–119.
26. Cazzulo-Klepzig, M., Mendonça-Filho, J.G., Peralba, M.C.R., Jasper, A., 1999. Caracterização do carvão da localidade de Quitéria, Encruzilhada do Sul, RS através de métodos palinológicos, petrográficos e organogeoquímicos. In: 51ª Reunião Nacional da Sociedade Brasileira para o Progresso da Ciência, Porto Alegre. Anais da Academia Brasileira de Ciências–Volume Especial. Rio de Janeiro: Academia Brasileira de Ciências, 71, 812–820.
27. Jasper, A., Menegat, R., Guerra-Sommer, M., Cazzulo-Klepzig, M., Souza, P.A., 2006. Depositional cyclicity and paleoecological variability in an outcrop of Rio Bonito formation, Early Permian, Paraná Basin, Rio Grande do Sul, Brazil. Journal of South American Earth Sciences, 21, 276–293.
28. Jasper, A., Uhl, D., Guerra-Sommer, M., Mosbrugger, V., 2008. Palaeobotanical evidence of wildfires in the late Palaeozoic of South America – Early Permian, Rio Bonito Formation, Paraná basin, Rio Grande do Sul, Brazil. Journal of South American Earth Sciences, 26(4), 435–444.
29. Guerra-Sommer, M., Cazzulo-Klepzig, M., Jasper, A., Kalkreuth, W., Menegat, R., Barboza, E.G., 2008a. Paleoecological patterns at the coal-roof shale transition in na outcrop of the Permian Brazilian Gondwana. Revista Brasileira de Paleontologia, 11, 11–26.
30. Costa, O.L., Kionka, D.C.O., Périco, E., Jasper, A., 2016. Identificação de carvão vegetal macroscópico no nível de roof-shale do Afloramento Quitéria, Formação Rio Bonito, Permiano Inferior da Bacia do Paraná. Geosul, 31, 133–154.
31. El Atfy, H., Havlik, P., Krüger, P. S., Manfroi, J., Jasper, A., Uhl, D., *in press*. Pre-Quaternary wood decay ‘caught in the act’by fire–examples of plant-microbe-interactions preserved in charcoal from clastic sediments. Historical Biology, 1–10.
32. Mendonça Filho, J.G., Sommer, M.G., Klepzig, M.C., Mendonça, J.O., Silva, T.F., Kern, M.L., Menezes, T.R., Jasper, A., Silva, M.C., Santos, L.G.C., 2013. Permian carbonaceous rocks from the Bonito Coalfield, Santa Catarina, Brazil: organic facies approaches. International Journal of Coal Geology, 111, 23–36.
33. Manfroi, J., Jasper, A., Guerra-Sommer, M., Uhl, D., 2012. Sub-arborescent lycophytes in coal bearing strata from the Artinskian (Early Permian/Cisuralian) of the Santa Catarina coalfield (Paraná Basin, SC, Brazil). Revista Brasileira Paleontologia, 15, 135–140.
34. Glasspool, I., 2003a. Palaeoecology of selected South African export coals from the Vryheid Formation, with emphasis on the role of heterosporous lycopods and wildfire derived inertinite. Fuel, 82, 959–970.
35. Glasspool, I., 2003b. Hypautochthonous-allochthonous coal deposition in the Permian, South African,Witbank Basin No. 2 seam; a combined approach using sedimentology, coal petrology and palaeontology. International Journal of Coal Geology, 53, 81–135.
36. Mahesh, S., Murthy, S., Chakraborty, B., Roy, M. D., 2015. Fossil charcoal as palaeofire indicators: taphonomy and morphology of charcoal remains in Sub-Surface Gondwana Sediments of South Karanpura Coalfield. Journal of the Geological Society of India, 85, 1-10.
37. Jasper, A., Agnihotri, D., Tewari, R., Spiekermann, R., Pires, E.F., Da Rosa, Á.A.S., Uhl, D., 2017. Fires in the mire: Repeated fire events in Early Permian ‘peat forming’ vegetation of India. Geological Journal, 52(6), 955–969.
38. Seetharam, D. S., Ramakrishna, H., 2017. Permian palaeo-wildfire on Gondwana land: charcoal remains from the Yellandu coalfield, Godavari graben, Telangana State, India. Environment and Ecology, 35(1), 93–96.
39. Mishra, S., Jha, N., 2017. Early Permian (Asselian–Sakmarian) palynoflora from Chintalapudi area, Godavari Graben, south India and its palaeoenvironmental implications. Journal of the Palaeontological Society of India, 62(2), 157–174.
40. Glasspool, I.J., and Scott, A.C. 2013. “Identifying past fire events,” in Fire phenomena and the Earth System: An Interdisciplinary Guide to Fire Science, ed. C.M. Belcher (Chichester: John Wiley and Sons, Ltd.), 179–205.
41. Gand, G., Galtier, J., Garric, J., Teboul, P.A., Pellenard, P., 2013. Discovery of an Autunian macroflora and lithostratigraphic re-investigation on the western border of the Lodéve Permian Basin (Mont Sénégra, Hérault, France). Paleoenvironmental implications. Comptes Rendus Palevol, 12(2), 69–79.
42. LePage, B.A., Beauchamp, B., Pfefferkorn, H.W., Utting, J., 2003. Late Early Permian plant fossils from the Canadian High Arctic: a rare paleoenvironmental/climatic window in northwest Pangea. Palaeogeography, Palaeoclimatology, Palaeoecology, 191, 345–372.
