# Supplementary material for: Recurrent palaeo-wildfires in a Cisuralian coal seam: A palaeobotanical view on high-inertinite coals from the Lower Permian of the Paraná Basin, Brazil
Source: PLoS One. 2019 Mar 14;14(3):e0213854. doi: 10.1371/journal.pone.0213854 (PMC6417680; doi:10.1371/journal.pone.0213854)
Supplement: S3 Table — Data based on [1,2,3,4,5], and additional sources not mentioned in these previous compilations. (DOCX) [file pone.0213854.s006.docx]

**S3 Table.** Published records of inertinites in Lower Permian coals. Data based on [1,2,3,4,5], and additional sources not mentioned in these previous compilations.

| Locality | Basin | Country | Age | References |
| --- | --- | --- | --- | --- |
| Pfälzer Bergland | Saar-Nahe Basin | Germany | Asselian | [6] |
| Dresden | Döhlener Basin | Germany | Asselian | [7] |
| Edwards county, Texas | Kerr Basin | United States | Asselian | [8] |
| Hedong coalfield | Ordos Basin | China | Asselian | [9] |
| Junger coalfield | Ordos Basin | China | Asselian | [10] |
| Jungar coalfield, Inner Mongolia | Ordos Basin | China | Asselian | [11,12] |
| Adaohai Mine, Daqingshan Coalfield, Inner Mongolia | Ordos Basin | China | Asselian | [13] |
| Jiangsu Province (Xuzhou) | - - - - - | China | Asselian-Sakmarian | [14] |
| Inner Mongolia (Zhungeer) | Ordos Basin | China | Asselian-Sakmarian | [14] |
| Shanxi Province (Pubai) | Ordos Basin | China | Asselian-Sakmarian | [14] |
| Hebei Province (Kaiping) | Ordos Basin? | China | Asselian-Sakmarian | [14,15] |
| Shanxi Province (Datong) | Ordos Basin | China | Asselian-Sakmarian | [16,17,14] |
| Shandong Province, Jining Coalfield | Ordos Basin | China | Asselian-Sakmarian | [18,19,14] |
| Shanxi Province, Gujiao CBM Block | Ordos Basin | China | Asselian-Sakmarian | [20] |
| Shuangliu coal mine | Ordos Basin | China | Asselian-Sakmarian | [21] |
| Shaanxi province, Hancheng | Ordos Basin | China | Asselian-Sakmarian | [22] |
| Wankie coalfield | Karoo Basin | Zimbabwe | Sakmarian | [23] |
| Son Valley coalfield | Son Valley Basin | India | Sakmarian | [24] |
| West Bakaro Coalfield | Damodar Valley Basin | India | Sakmarian | [25] |
| Auranga Coalfield | Saptura Basin | India | Sakmarian | [26] |
| Candiota coal mine | Paraná Basin | Brazil | Sakmarian | [27,28,29] |
| Leão-Butiá | Paraná Basin | Brazil | Sakmarian | [29] |
| Santa Terezinha | Paraná Basin | Brazil | Sakmarian | [29] |
| Bonito coal mine | Paraná Basin | Brazil | Artinskian | [30] |
| Porongos outcrop | Paraná Basin | Brazil | Artinskian | This work |
| Novo Horizonte coal mine | Paraná Basin | Brazil | Artinskian | [30] |
| Esperança coal mine | Paraná Basin | Brazil | Artinskian | [30] |
| Fontanela coal mine | Paraná Basin | Brazil | Artinskian | [30] |
| Morrizini coal mine | Paraná Basin | Brazil | Artinskian | [30] |
| Malha II Norte coal mine | Paraná Basin | Brazil | Artinskian | [30] |
| Do Trevo coal mine | Paraná Basin | Brazil | Artinskian | [30] |
| São Simão coal mine | Paraná Basin | Brazil | Artinskian | [30] |

(continued)

| Locality | Basin | Country | Age | References |
| --- | --- | --- | --- | --- |
| Companhia Criciúma - Verdinho coal mine | Paraná Basin | Brazil | Artinskian | [30 |
| Companhia Cooperminas - Verdinho coal mine | Paraná Basin | Brazil | Artinskian | [30]) |
| Mineira II coal mine | Paraná Basin | Brazil | Artinskian | [30] |
| Maracajá, South, Santa Catarina | Paraná Basin | Brazil | Artinskian | [31] |
| Cruz de Malta coal mine | Paraná Basin | Brazil | Artinskian | [32] |
| Lauro Müller coal mine | Paraná Basin | Brazil | Artinskian | [32] |
| Figueira coalfield | Paraná Basin | Brazil | Sakmarian-Artinskian | [33] |
| Sakamena | Houille Malgache Basin | Madagascar | Sakmarian-Artinskian | [34] |
| Greta Coal Measures | Sydney Basin | Australia | Artinskian | [35,36,37] |
| Patchawarra Formation | Cooper Basin | Australia | Lower Permian | [38] |
| Irwin River Coal | Perth Basin | Australia | Artinskian | [39] |
| Ewington coal mine | Perth Basin | Australia | Artinskian | [39] |
| Sue coal mine, Vasse shelf | Perth Basin | Australia | Artinskian | [39,40] |
| Ashford Coal Mine | Ashford Basin | Australia | Artinskian | [41] |
| Johilla Coalfield | South Rewa Gondwana Basin | India | Artinskian | [42] |
| Main S. Gwembe | Mid-Zambezi Valley Basin | Zambia | Artinskian | [43] |
| Pench | Saptura Basin | India | Artinskian | [44] |
| Kanhan | Saptura Basin | India | Artinskian | [44] |
| Tawa Valley | Saptura Basin | India | Artinskian | [44] |
| Bhangtar area, district of Samdrup Jongkhar | Bhutan Basin | Bhutan | Artinskian | [45] |
| Southeastern Bhutan | Bhutan Basin | Bhutan | Artinskian | [46] |
| Ogies region | Karoo Basin | Africa | Artinskian | [47] |
| Spitzkop mine | Karoo Basin | Africa | Artinskian | [48] |
| Middelburg mine | Karoo Basin | Africa | Artinskian | [48] |
| Rietspruit mine | Karoo Basin | Africa | Artinskian | [48] |
| Goedehoop mine | Karoo Basin | Africa | Artinskian | [48] |
| Kromdraai mine | Karoo Basin | Africa | Artinskian | [48] |
| Kleinkopje mine | Karoo Basin | Africa | Artinskian | [48] |
| Petchora | Petchora Coal Basin | Russia | Artinskian-Kungurian | [49] |
| Jamalganj coalfield | Jamalganj Basin | Bangladesh | Artinskian-Kungurian | [50] |
|  |  |  |  |  |

(continued)

| Locality | Basin | Country | Age | References |
| --- | --- | --- | --- | --- |
| Collinsville coal measures | Bowen Basin | Australia | Artinskian-Kungurian | [51,52] |
| Moatize | Karoo Basin | Mozambique | Artinskian - Kungurian | [53] |
| Mucanha - Vuzi | Karoo Basin | Mozambique | Artinskian - Kungurian | [54] |
| East of Guizhou Province | - - - - - | China | Artinskian–Kungurian | [15] |
|  |  |  |  |  |
| East of Yunnan Province (Huize, Zhanyi) | - - - - - | China | Artinskian–Kungurian | [15] |
|  |  |  |  |  |
| West of Hunan Province (Huaihua, Xupu) | - - - - - | China | Artinskian–Kungurian | [14,15] |
| North of Jiangxi Province (Xiushui) | - - - - - | China | Artinskian–Kungurian | [15] |
|  |  |  |  |  |
| South of Hubei Province (Jianshi, Puqi, Yangxin) | - - - - - | China | Artinskian–Kungurian | [15] |
| Henan Province (Jiaozuo, Pingdingshan, Mianchi) | - - - - - | China | Artinskian–Kungurian | [14,15] |
|  |  |  |  |  |
| Ningxia Province (Shizuishan) | Ordos Basin | China | Artinskian–Kungurian | [14,15] |
|  |  |  |  |  |
| Inner Mongolia (Qingshuihe, Wuda, Zhungeer) | Ordos Basin | China | Artinskian–Kungurian | [14,15] |
|  |  |  |  |  |
| Shaanxi Province (Pubai, Fugu, Tongchuan, Pubai, Chengcheng) | Ordos Basin | China | Artinskian–Kungurian | [14,15] |
| Shanxi Province | Ordos Basin | China | Artinskian–Kungurian | [55,15,16,17,14] |
| Hebei Province (Fengfeng, Xingtai, Lincheng, Tangshan, Dacheng, Kaiping), Xingtai Coalfield, Hebei province, northern China | Ordos Basin | China | Artinskian–Kungurian | [14,15,56,57] |
|  |  |  |  |  |
| West Shandong Province | - - - - - | China | Artinskian–Kungurian | [14,15,18,19] |
| Jiangsu Province (Xuzhou) | North China Basin | China | Artinskian–Kungurian | [14,15] |
| Liaoning Province | North China Basin | China | Artinskian–Kungurian | [14,15] |
| Anhui Province (Huainan, Huaibei) | North China Basin | China | Artinskian–Kungurian | [14,15] |
| West Papua Province | Papuan Basin? | Indonesia | Artinskian - Kungurian | [58] |
| Pench, Kanhan, and Tawa Valley Coalfields | Saptura Basin | India | Artinskian - Kungurian | [59,26,60,44] |
| Singrauli coalfield | Goodavari Valley Basin | India | Artinskian - Kungurian | [61] |
| Boreholes (TS-28, SPS- 1, NBW- 17, BKS- 17, GD-33, AP-74 and DB-31 ) | Damodar Valley Basin | India | Artinskian - Kungurian | [62] |
| Kusunda coal mine | Damodar Valley Basin | India | Artinskian - Kungurian | [62] |
| Moonidih coal mine | Damodar Valley Basin | India | Artinskian - Kungurian | [62] |

(continued)

| Locality | Basin | Country | Age | References |
| --- | --- | --- | --- | --- |
| Coal seams of Sawang Colliery, Jharkhand | Damodar Valley Basin | India | Artinskian - Kungurian | [63] |
| Namwele-Mkomolo coalfield | Rukwa Basin | Tanzania | Artinskian - Kungurian | [64] |
| Muze coalfield | Rukwa Basin | Tanzania | Artinskian - Kungurian | [64] |
| Galula coalfield | Rukwa Basin | Tanzania | Artinskian - Kungurian | [64] |
| New South Wales | Gunnedah  Basin | Australia | Artinskian - Kungurian | [60] |
| Songwe – Kiwira coalfield | Karoo Basin | Tanzania | Artinskian - Kungurian | [65] |
| Northern Province | Karoo Basin | South Africa | Artinskian - Kungurian | [66] |
| Mpulanga Province | Karoo Basin | South Africa | Artinskian - Kungurian | [66] |
| KwaZulu | Karoo Basin | South Africa | Artinskian - Kungurian | [66] |
| Wankie coalfield | Karoo Basin | Zimbabwe | Kungurian | [23,67,68] |
| Sabi | Sabi-Lundi Coal Basin | Zimbabwe | Kungurian | [68] |
| Witbank coalfield | Karoo Basin | South Africa | Kungurian | [69] |
| Highveld coalfield | Karoo Basin | South Africa | Kungurian | [70,71] |
| Hazaribagh District | Goodavari Valley Basin | India | Kungurian | [72] |
| Ramagundam coalfields | Goodavari Valley Basin | India | Kungurian | [73] |
| Kothagudem coalfields | Goodavari Valley Basin | India | Kungurian | [73] |
| West and East Bokaro coalfield | Damodar Valley Basin | India | Kungurian | [25] |
| Dighipara | Dighipara Basin | Bangladesh | Lower Permian | [74] |
| Queensland | Bowen Basin | Australia | Kungurian | [75] |
| Komi Republic (Pechorsky, Vorkutskoye, Khal'mer-Yuskoy, Yun'-Yaginskoye, Moschny Troinoy Districts, Vorkuta) | Pechora Coal Basin | Russia | Kungurian | [76,77] |
| Kemerovskaya, Dvoinoy and Podsporny Districts | Petchora Coal Basin | Russia | Kungurian | [77] |

**References**

1. Diessel, C.F., 2010. The stratigraphic distribution of inertinite. International Journal of Coal Geology, 81(4), 251–268.
2. Glasspool, I.J., Scott, A.C., 2010. Phanerozoic concentrations of atmospheric oxygen reconstructed from sedimentary charcoal. Nature Geosciences, 3, 627–630.
3. Jasper, A., Guerra-Sommer, M., Hamad, A.M.A., Bamford, M., Bernardes-de-Oliveira, M.E.C., Tewari, R., Uhl, D., 2013. The burning of Gondwana: Permian fires on the southern continent–a palaeobotanical approach. Gondwana Research, 24(1), 148–160.
4. Abu Hamad, A.M.A., Jasper, A., Uhl, D., 2012. The record of Triassic charcoal and other evidence for palaeo-wildfires: signal for atmospheric oxygen levels, taphonomic biases or lack of fuel. International Journal of Coal Geology, 96, 60–71.
5. Yan, M., Wan, M., He, X., Hou, X., Wang, J., 2016. First report of Cisuralian (Early Permian) charcoal layers within a coal bed from Baode, North China with reference to global wildfire distribution. Palaeogeography, Palaeoclimatology, Palaeoecology, 459, 394–408.
6. Josten, K.-H., 1956. Die Kohlen im Pfälzer Bergland. Notizblätter des Hessischen Landesamtes für Bodenforschung, 84, 300–327.
7. Christoph, H.J., 1957. Qualitative und quantitative kohlenpetrographische Untersuchungen des Hauptflözes des Döhlener Beckens. Freiberger Forschungshefte, 37, 22–41.
8. Barker, C., Heck, W., Eble, C., 2003. Coalbed methane in Late Pennsylvanian to Early Permian coal, Kerr Basin, Edwards County, Texas. Gulf Coast Association of Geological Societies 53rd Annual Convention, Baton Rouge, LA, Abstracts.
9. Li, B.-F., Wen, X.-D., Kang, X.-D., Li, G.-D., 1997. The applications of high resolution sequence stratigraphy to paralic and terrestrial coal-bearing strata–two case studies from the western North China Paleozoic basin and the Tulufan-Hami Jurassic Basin. Proceedings 30th International Geological Congress Part B, 1–19.
10. Dai, S., Ren, D., Chou, C.-L., Li, S., Jiang, Y., 2006. Mineralogy and geochemistry of the No. 6 coal (Pennsylvanian) in the Junger Coalfield, Ordos Basin, China. International Journal of Coal Geology, 66, 253–270.
11. Dai, S., Li, D., Chou, C.-L., Zhao, L., Zhang, Y., Ren, D., Ma, Y., Sun, Y., 2008. Mineralogy and geochemistry of boehmite-rich coals: new insights from the Haerwusu Surface Mine, Jungar Coalfield, Inner Mongolia, China. International Journal of Coal Geology 74, 185–202.
12. Dai, S., Jiang, Y., Ward, C.R., Gu, L., Seredin, V.V., Liu, H., Zhou, D., Wang, X., Sun, Y., Zou, J., Ren, D., 2012a. Mineralogical and geochemical compositions of the coal in the Guanbanwusu Mine, Inner Mongolia, China: further evidence for the existence of an Al (Ga and REE) ore deposit in the Jungar Coalfield. International Journal of Coal Geology, 98, 10–40
13. Dai, S., Zou, J., Jiang, Y., Ward, C.R., Wang, X., Li, T., Xue, W., Liu, S., Tian, H., Sun, X., Zhou, D., 2012b. Mineralogical and geochemical compositions of the Pennsylvanian coal in the Adaohai Mine, Daqingshan Coalfield, Inner Mongolia, China: modes of occurrence and origin of diaspore, gorceixite, and ammonian illite. International Journal of Coal Geology, 94, 250–270.
14. Yang, Y., Zou, R., Shi, Z., Jiang, R., 1996. Atlas for Coal Petrography of China. China Univ. Mining and Technology Press, Xuzhou in Chinese.
15. Han, D., Ren, D., Wang, Y., Jin, K, Mao, H., Qin, Y., 1996. Coal Petrology of China. China University of Mining and Technology Press, Xuzhou in Chinese.
16. IGE, (Institute of Geology Exploration CCMRI Coal Ministry), PCEC, (Provincial Coalfields Exploration Corporation of Shanxi), 1987a. Sedimentary Environment of the Coalbearing Strata in Pinglu-Shuoxing Mining Area, China. Shaanxi People's Education Publishing House, Xi'an in Chinese.
17. IGE, (Institute of Geology Exploration CCMRI Coal Ministry), PCEC, (Provincial Coalfields Exploration Corporation of Shanxi), 1987b. Sedimentary Environments of Taiyuan Xishan Coal Basin. China Coal Industry Publishing House, Beijing in Chinese.
18. Liu, G.J., Yang, P.Y., Peng, Z.C., Chou, C.L., 2004. Petrographic and geochemical contrasts and environmentally significant trace elements in marine-influenced coal seams, Yanzhou mining area, China. Journal of Asian Earth Sciences, 23, 491–506.
19. Querol, X., Alastuey, A., Lopez-Soler, A., Plana, F., Zeng, R., Zhao, J., Zhuang, X., 1999. Geological controls on the quality of coals from the West Shandong mining district, Eastern China International Journal of Coal Geology, 42, 63–88.
20. Zhao, L., Qin, Y., Cai, C., Xie, Y., Wang, G., Huang, B., Xu, C., 2017. Control of coal facies to adsorption-desorption divergence of coals: A case from the Xiqu Drainage Area, Gujiao CBM Block, North China. International Journal of Coal Geology, 171, 169–184.
21. Yang, N., Tang, S. H., Zhang, S. H., Xi, Z. D., Li, J., Yuan, Y., Guo, Y. Y., 2018. In seam variation of element-oxides and trace elements in coal from the eastern Ordos Basin, China. International Journal of Coal Geology, 197, 31–41.
22. Zhao, J., Tang, D., Qin, Y., Xu, H., 2018. Experimental study on structural models of coal macrolithotypes and its well logging responses in the Hancheng area, Ordos Basin, China. Journal of Petroleum Science and Engineering, 166, 658–672.
23. Watson, R., 1958. The origin of Wankie coal. South African Journal of Geology, 61, 1–12.
24. Basu, T., 1967. Petrographic characteristics and their bearing on the origin and correlation of Indian coals. J. Mines Met. Fuels, 15, 177–186.
25. Navale, G., Saxena, R., 1989. An appraisal of coal petrographic facies in Lower Gondwana (Permian) coal seams of India. International Journal of Coal Geology, 12, 553–588.
26. Jha, B.R., Jha, G., 1996. Petro-palynological studies of Permian coals from Jagaldagga sector of Auranga Coalfield, District Palamau, Bihar, India. In: Ayyasami, K., Sengupta, S., Ghosh, R.N. (Eds.), Gondwana Nine–Proceedings of the Ninth International Gondwana Symposium, Hyderabad, India, January 1994. Oxford and IBH, New Delhi, 89–108.
27. Ade, M.V.B., Silva, M.B., Corrêa da Silva, Z.C., 1998. Palaeoenvironments of coal seams deposition in Candiota Coalfield, South Brazil, based on maceral composition, Faculdade de Sciências do Porto, vol. 5. In: Lemos de Sousa, M.J., Marques, M.M., Fernandes, J.P. (Eds.), 2nd Symposium on Gondwana Coals, Porto 1998, Departamento de Geologia, Memória, 13–24.
28. Silva, M.B., Kalkreuth, W., 2005. Petrological and geochemical characterization of Candiota coal seams, Brazil–implication for coal facies interpretations and coal rank. International Journal of Coal Geology, 64, 217–238.
29. Kalkreuth, W.D., Holz, M., Kern, M., Machado, G., Mexias, A., Silva, M.B., Willett, J., Finkelman, R., Burger, H., 2006. Petrology and chemistry of Permian coals from the Paraná Basin: 1. Santa Terezinha, Leão-Butiá and Candiota Coalfields, Rio Grande do Sul, Brazil. International Journal of Coal Geology, 68, 79–116.
30. Kalkreuth, W., Holz, M., Mexias, A., Balbinot, M., Levandowski, J., Willett, J., Finkelman, R., Burger, H., 2010. Depositional setting, petrology and geochemistry of Permian coals from the Paraná Basin: 2. South Santa Catarina Coalfield, Brazil. International Journal of Coal Geology, 84, 213–236.
31. Da Costa, J.B., Lourenzi, P.D.S., González, M.B., do CR Peralba, M., Kalkreuth, W., 2014. A petrological and organic geochemical study of Permian coal seams east of Maracaja, South Santa Catarina, Parana Basin, Brazil. International Journal of Coal Geology, 132, 51–59.
32. Simão, G., Kalkreuth, W., 2015. Petrographic and chemical characterization of the Bonito Seam and its beneficiation products, South Santa Catarina Coalfield–Brazil. Energy Exploration & Exploitation, 33(1), 75–90.
33. Ricardi-Branco, F., Fernandes, J.P., Flores, D., 1998. Organic petrology and palynology of coals from Figueira (Paraná, Brazil), Faculdade de Sciências do Porto, vol. 5. In: Lemos de Sousa, M.J., Marques, M.M., Fernandes, J.P. (Eds.), 2nd Symposium on Gondwana Coals, Porto 1998. Departamento de Geologia, Memória, 169–172.
34. Alpern, B., Rakotoarivelo, H., 1972. Étude pétrographie du bassin houiller malgache. Annales de la Société Géologique du Nord (Lille), 92, 67–74.
35. Edwards, G.E., 1975. Marketable resources of Australian coal. The Australasian Institute of Mining and Metallurgy, 85–108.
36. Diessel, C.F.K., Gammidge, L.C., 2003. Downhole vitrinite reflectance in DM Tangorin DDH1. Coal and petroleum bulletin, vol. 4. In: Facer, R.A., Foster, C.B. (Eds.), Geology of the Cranky Corner Basin. New South Wales Department of Mineral Resources, 107–114.
37. Izart, A., Suarez-Ruiz, I., Bailey, J., 2015. Paleoclimate reconstruction frompetrography and biomarker geochemistry from Permian humic coals in Sydney Coal Basin (Australia). International Journal of Coal Geology, 138, 145–157.
38. Smyth, M., 1984. Coal microlithotypes related to sedimentary environments in the Cooper Basin, Australia. Internation Association of Sedimentologists Special Publications, 7, 333–347.
39. Santoso, B., 1994. Petrology of Permian coal, Vasse Shelf, Perth Basin, Western Australia. Ph.D. Thesis, Curtin University of Technology, W. A.
40. Le Blanc Smith, G., Mory, A., 1995. Geology and Permian coal resources of the Irwin Terrace. Perth Basin, Western Australia: Geological Survey of Western Australia, Report, 44, 1–60.
41. Flood, P.G., 1995. Asford area, NSW. In: Ward, C.R., Harrington, H.J., Mallet, C.W., Beeston, J.W. (Eds.), Geology of Australian Coal Basins. Coal Geology Group, Spec. Publ., 1. Geological Society of Australia Inc., 07–409.
42. Singh, R.M., Sing, K.N., 1987. The petrology of Nowrozabad and Birsinghpur coals, Johilla Coalfield, Son Valley. National Seminar on Coal Resources of India, Proceedings, 293–323.
43. Money, N.J., Drysdall, A.R., 1973. The geology, classification, palaeogeography and origin of the Mid-Zambesi Coal deposits of Zambia. In: Campbell, K.S.W. (Ed.), Gondwana Geology–Papers from the Third Gondwana Symposium Canberra, 1973. Australian National University Press, 249–270.
44. Singh, M.P., Shukla, R.R., 2004. Petrographic characteristics and depositional conditions of Permian coals of Pench, Kanhan, and Tawa Valley Coalfields of Saptura Basin, Madhya Pradesh, India. International Journal of Coal Geology, 59, 209–243.
45. Pareek, H.S., 1990. Petrography and rank of the Bhangtar coals, southeastern Bhutan. International Journal of Coal Geology, 15, 219–243.
46. Mukherjee, A.K., Alam, M.M., Ghose, S., 1988. Gondwana coals of Bhutan Himalaya– occurrence, properties, and petrographic characteristics. International Journal of Coal Geology, 9, 287–304.
47. Glasspool, I., 2003a. Palaeoecology of selected South African export coals from the Vryheid Formation, with emphasis on the role of heterosporous lycopods and wildfire derived inertinite. Fuel, 82, 959–970.
48. Glasspool, I., 2003b. Hypautochthonous–allochthonous coal deposition in the Permian, South African, Witbank Basin No. 2 seam; a combined approach using sedimentology, coal petrology and palaeontology. International Journal of Coal Geology, 53, 81–135.
49. Volkova, I.B., 1986. The petrography of the coals of the USSR. An inventory of the petrographic composition of the coal basins of the USSR. Inst. Geol. A.P. Karpinskovo. N.S., T., 333. 248 pp. (in Russian).
50. Imam, M.B., Rahman, M., Akhter, S.H., 2002. Coalbed methane prospect of Jamalganj Coalfield, Bangladesh. Arabian Journal for Science and Engineering, 27, 17–27.
51. Beeston, J.W., Davis, A., 1976. Petrography of coal seams in Departmental Borehole Bowen N.S. 597, Collinsville. Queensland Government Mining Journal, 1–11.
52. Mutton, A.J. (Compiler), 2003. Queensland Coals 14th Edition. Queensland Department of Natural Resources and Mines, 1–112.
53. Annon, 1983. Investigations of coal samples taken under the Moatize (II) exploration Programme to evaluate their material composition, raw material and coking properties for characterisation of the examined deposit areas. Brennstoffinstitut Freiberg-DDR, November 1983, unpublished report.
54. Falcon, R.M.S., Sousa, M.J.L., Pinheiro, H.J., Marques, M.M., 1984. Petrology and palynology of Mozambique coals–Mucanha-Vúzi Region. Comunicações dos Serviços Geológicos de Portugal Lisboa, 70, 321–338.
55. Cheng, B.Z., 1992. Late Paleozoic Sedimentary Environments and Coal Accumulation in Shanxi. Shanxi Science and Technology Press, Taiyuan in Chinese.
56. Sun, Y., Püttmann, W., Kalkreuth, W., Horsfield, B., 2002. Petrologic and geochemical characteristics of Seam 9–3 and Seam 2, Xingtai Coalfield, Northern China. International Journal of Coal Geology, 49, 251–262.
57. Sun, Y., Zhao, C., Zhang, J., Yang, J., Zhang, Y., Yuan, Y., Duan, D., 2013. Concentrations of valuable elements of the coals from the Pingshuo Mining District, Ningwu Coalfield, northern China. Energy Exploration & Exploitation, 31(5), 727-744.
58. Belkin, H.E., Tewalt, S.J., Hower, J.C., Stucker, J.D., O'Keefe, J.M.K., 2009. Geochemistry and petrology of selected coal samples from Sumatra, Kalimantan, Sulawesi, and Papua, Indonesia. International Journal of Coal Geology, 77, 260–268.
59. Chakrabarti, N.C., 1987. Petrography and rank in relation to coking characters of certain coal seams in Sohagpur coalfield, Madhya Pradesh. National Seminar on Coal Resources India. Proceedings, 244–263.
60. Gurba, L.W., Ward, C.R., 2000. Elemental composition of coal macerals in relation tovitrinite reflectance, Gunnedah Basin, Australia, as determined by electron microprobe analysis. International Journal of Coal Geology, 44, 127–147.
61. Misra, B.K., Singh, B.D., 1990. The Lower Permian coal seams from Singrauli coalfield (M.P.), India: petrochemical nature, rank, age and sedimentation. International Journal of Coal Geology, 24, 309–342.
62. Mishra, H.K., Cook, A.C., 1992. Petrology and thermal maturity of coals in the Jharia Basin: implications for oil and gas origins. International Journal of Coal Geology, 20, 277–313.
63. Pophare, A.M., Mendhe, V.A., Varade, A., 2008. Evaluation of coal bed methane potential of coal seams of Sawang Colliery, Jharkhand, India. Journal of Earth System Science, 117, 121–132.
64. Semkiwa, P., Kalkreuth, W., Utting, J., Mayagilo, F., Mpanju, F., Hagemann, H., 1998. The geology, petrology, palynology and geochemistry of Permian coal basins in Tanzania. 1. Namwele–Mkomolo, Muze asnd Galula coalfields. International Journal of Coal Geology, 36, 63–110.
65. Semkiwa, P., Kalkreuth, W., Utting, J., Mpanju, F., Hagemann, H., 2003. The geology, petrology, palynology and geochemistry of Permian coal basins in Tanzania 2. Songwe-Kiwira Coalfield. International Journal of Coal Geology, 55, 157–186.
66. Fabiańska, M.J., Kruszewska, K.K.J., 2003. Relationship between petrographic and geochemical characterisation of selected South African coals. International Journal of Coal Geology, 54, 95–114.
67. Duguid, K.B.B., 1978. The Geology of the Karoo System in the Sabi and Lundi–Sabi Basins. Department of Geology, University of Rhodesia, 256–316.
68. Carr, A.D., Williamson, J.E., 1990. The relationship between aromaticity, vitrinite reflectance and maceral composition of coals: implications for the use of vitrinite reflectance as a maturation parameter. Organic Geochemistry, 16, 313–323.
69. Mangena, S.J., Korte, G.J., McGrindle, R.I., Morgan, D.L., 2004. The amenability of some Witbank bituminous ultra-fine coals to binderless briquetting. Fuel Processing Technology, 85, 1647–1662.
70. Hagelskamp, H.H.B., Snyman, C.P., 1988. On the origin of low-reflecting inertinites in coals from the Highveld Coalfield, South Africa. Fuel, 67, 307–313.
71. Wagner, N.J., Hlatshwayo, B., 2005. The occurrence of potentially hazardous trace elements in five Highveld coals, South Africa. International Journal of Coal Geology, 63, 228–246.
72. Pareek, H.S., Bardhan, B., 1985. Trace elements and their variation along seam profiles of the Middle and Upper Barakar formations (Lower Permian) in the East Bokaro coalfield, district Hazaribagh, Bihar, India. International Journal of Coal Geology, 5, 281–314.
73. Pareek, H.S., 1986. The role of coal petrographic characteristics in evaluating the noncoking nature of the coals of Ramagundam and Kothagudem coalfields, Goodavari Valley basins, Andhra Pradesh, India. International Journal of Coal Geology, 6, 181–198.
74. Farhaduzzaman, M., Abdullah, W.H., Islam, M.A., Sia, S.G., 2015. Quality Assessment of the Permian Coals from Dighipara Basin, Bangladesh Based On Proximate, Ultimate and Microscopic Analyses. Journal of Bangladesh Academy of Sciences, 39(2), 177–194.
75. Follington, I.L., Beeston, J.W., Hamilton, L.H., 1995. Bowen Basin Symposium 1995–150 Years on, Geological Society Australia Coal Geology Group, Brisbane, 1–472.
76. Sallabasheva, V., 1979. Petrological study of coal from the metallurgical works “Kremikovcki”. Annales Universitates Sofia, 71 (1), 323–337.
77. Brownfield, M.E., Steinshouer, D.W., Povarennykh, M.Yu, Eriomin, I., Shpirt, M., Meitov, Y., Sharova, I., Goriunova, N., Zyrianova, M.V., 2001. Coal quality and resources of the former Soviet Union–an ArcView project. U.S. Geol. Surv. Open-File Report, 01, 1–94.
